# Genetic Associations Among Appendicular Lean Mass, Alanine Aminotransferase, and Type 2 Diabetes: Multi-Dataset Mendelian Randomization and Colocalization at the *SERPINA1* Candidate Locus

**DOI:** 10.3390/genes17091154

**Published:** 2026-09-20

**Authors:** Xinyuan Wang, Wenchuan Yang, Shuhua Song, Huaiyi Su, Yu Zhang

**Affiliations:** School of Physical Education, Yunnan Normal University, 768 Juxian Street, Chenggong District, Kunming 650500, China; xinyuanw908@gmail.com (X.W.); ywc18118648951@163.com (W.Y.); 15085308749@163.com (H.S.); 18408741129@163.com (Y.Z.)

**Keywords:** appendicular lean mass, alanine aminotransferase, type 2 diabetes, Mendelian randomization, colocalization, *SERPINA1*

## Abstract

**Background/Objectives:** Appendicular lean mass (ALM), alanine aminotransferase (ALT), and type 2 diabetes (T2D) may be genetically linked. However, the ALT-related pathway and shared signals at the *SERPINA1* candidate locus remain uncertain. We used Mendelian randomization (MR) to evaluate associations among ALM, ALT, and T2D and compared candidate-locus signals across datasets. ALM represented muscle mass, not clinical sarcopenia. **Methods:** Using European-ancestry GWAS summary statistics, we performed six bidirectional two-sample MR analyses, multivariable MR (MVMR) with finite-sample t-based inference, and exploratory path-specific two-step MR. FinnGen R12 provided the T2D outcome data for MR and pathway analyses. Pathway analyses used 5000 shared-sampling bootstrap replicates to assess uncertainty in path estimates. We combined locus-level coloc/ABF with signal-specific SuSiE-coloc in the GRCh37 *SERPINA1* region. T2DGGI was used only for colocalization. Molecular-QTL and external-omics analyses were treated as supportive or limiting evidence. **Results:** Higher genetically predicted ALM was associated with lower ALT and lower T2D risk, whereas higher genetically predicted ALT was associated with greater T2D risk. T2D→ALT was method-dependent. In MVMR, the ALT conditional effect remained positive, whereas the ALM direct effect was model-dependent. Path decomposition was compatible with an ALT-related statistical pathway, but product- and difference-based estimates disagreed, and residual heterogeneity remained. ALM–T2DGGI supported an rs28929474-related shared component, although component-cohort overlap was possible. FinnGen supported other ALT-related signals but did not independently corroborate rs28929474. Tissue eQTL and external omics did not provide consistent mechanistic support. **Conclusions:** The data support directionally connected MR-based associations and dataset-dependent shared candidate signals. These findings do not establish biological mediation, a unique causal variant, gene-level causal assignment, or a molecular mechanism.

## 1. Introduction

Sarcopenia is a progressive, aging-related skeletal-muscle disorder characterized primarily by reduced strength and assessed alongside muscle quantity or quality and physical performance. It is associated with falls, disability, and mortality [1,2,3,4]. A single genetic phenotype cannot capture all dimensions of clinical sarcopenia. Here, appendicular lean mass (ALM) represented muscle mass rather than clinical sarcopenia. Sarcopenia provided the clinical background and phenotypic context for the external skeletal-muscle transcriptome analyses.

Skeletal muscle is a major site of peripheral glucose uptake during insulin stimulation. Reduced muscle mass and impaired muscle function may be associated with insulin resistance, metabolic dysregulation, and higher risk of type 2 diabetes (T2D) [5,6,7,8]. Cohort and clinical studies have also reported associations between muscle-related phenotypes and T2D risk [9,10,11,12]. However, age, adiposity, comorbidity, residual confounding, and reverse causation make the direction of these associations difficult to establish [7,12,13,14]. Mendelian randomization (MR) uses exposure-associated genetic variants as instrumental variables. Under its core assumptions, MR can reduce some confounding and reverse-causation bias, providing evidence on the direction of the ALM–T2D association [13,14,15].

The liver contributes to systemic metabolic homeostasis through gluconeogenesis, lipid metabolism, and insulin clearance [16,17]. Alanine aminotransferase (ALT) reflects hepatocellular injury and is also associated with fatty liver, insulin resistance, and T2D risk [18,19,20]. These relationships suggest possible genetic links among ALM, ALT, and T2D. Genetically predicted higher ALM may be associated with lower ALT and lower T2D risk. Genetically predicted higher ALT may, in turn, be associated with higher T2D risk. However, pairwise associations cannot distinguish an ALT-related pathway from reverse effects or horizontal pleiotropy. Statistical decomposition alone also cannot establish biological mediation between these traits. These questions require joint consideration of bidirectional MR, robustness checks, multivariable MR (MVMR), and approximate indirect-effect analyses. MR studies across sex and ancestry also suggest heterogeneity in the genetic relationships of lean mass and fat mass with T2D [21]. Interpretation of our findings should therefore remain specific to the phenotypes and datasets analyzed here.

We selected *SERPINA1* as a candidate region based on prior evidence, rather than a genome-wide shared-locus scan. This choice was based on four considerations. First, the *SERPINA1* Pi*Z allele has been associated with abnormal liver enzymes, liver injury, and cirrhosis risk [22]. Second, the missense variant rs28929474 has been associated with a lower visceral-to-gluteofemoral fat ratio [23]. Third, its encoded protein, alpha-1 antitrypsin (AAT), is closely linked to *SERPINA1*-related hepatic phenotypes. Finally, preclinical experiments have linked AAT to glucose tolerance, insulin secretion, and beta-cell stress [24]. Together, these findings motivated examination of a possible regional link among fat distribution, hepatocellular injury, and glucose metabolism. We therefore used single-signal coloc/ABF and multi-signal SuSiE-coloc [25,26] to assess whether ALM, ALT, and T2D shared candidate signals within this region. We then integrated cis-eQTL, plasma cis-pQTL, transcriptomic, and network evidence to constrain their biological interpretation. However, regional colocalization alone cannot assign a shared signal to *SERPINA1* or identify a unique causal variant. It also cannot establish AAT as a mediator or therapeutic target. Our analysis therefore does not identify *SERPINA1* as the principal or only locus connecting these traits.

Previous studies have characterized the genetic architecture of ALM using UK Biobank data [27]. MR analyses have also reported associations between lean-mass-related or sarcopenia-related traits and T2D [28,29,30]. Our contribution extends beyond the pairwise ALM–T2D association by integrating six prespecified MR directions, MVMR, and exploratory decomposition of the ALM→ALT→T2D candidate pathway. We further compared candidate-region colocalization across T2D datasets using locus-level and signal-specific analyses. Molecular-QTL and external-omics evidence were used to test, rather than assume, a *SERPINA1*-based molecular explanation. This framework distinguishes genome-wide MR associations, dataset-dependent regional signal sharing, and biological mechanism.

## 2. Materials and Methods

### 2.1. Study Design and Data Sources

The study was reported according to the STROBE-MR principles [31]. A layered evidence framework was used to evaluate MR-based genetic associations among ALM, ALT, and T2D. The first layer comprised six bidirectional two-sample MR directions, MVMR, and exploratory path-specific two-step MR. The second layer comprised locus-level and signal-specific colocalization of the knowledge-driven *SERPINA1* candidate region, molecular-QTL colocalization, and external transcriptomic, gene-set, and STRING network analyses. Each evidence layer assessed trait direction, shared candidate signals, or external biological concordance. The evidence layers were not combined into a composite mechanistic score.

The primary analyses used European-ancestry GWAS summary statistics. The ALM dataset was ebi-a-GCST90000025 (*n* = 450,243) [27]. FinnGen R12 T2D included 82,878 cases and 403,489 controls [32]. ALT was obtained from the MVP Genomics Release 4 European-ancestry mean-ALT GWAS (ALT_Mean_INT.EUR.GIA; *n* = 406,803) [33]. BMI was obtained from ieu-b-40 (*n* = 681,275) [34]. MVP ALT was a continuous trait adjusted for age, sex, and principal components and subjected to inverse-normal transformation. Its beta coefficients were retained on the transformed GWAS scale. They cannot be interpreted as effects per 1 U/L increase in clinical ALT. BMI was used only in MVMR sensitivity analyses. FinnGen R12 was consistently used as the T2D outcome for MR, MVMR, and pathway analyses.

The candidate-region analyses used both FinnGen R12 and the European subgroup of the 2024 T2D Global Genomic Initiative (T2DGGI; 242,283 cases and 1,569,734 controls) [35]. T2DGGI was used only for colocalization, whereas FinnGen remained the MR outcome dataset. Effect estimates from the two datasets were not pooled. FinnGen provided cross-cohort sensitivity evidence, whereas T2DGGI provided a higher-powered regional comparison. T2DGGI may include component cohorts that overlap with other resources. Its results were therefore treated as supportive evidence rather than independent corroboration.

We qualitatively assessed potential participant overlap using documented cohort provenance (Supplementary Table S1c,d). Exact overlap counts could not be determined from the summary data. The ALM, ALT, and FinnGen T2D datasets originated from UK Biobank, the Million Veteran Program, and FinnGen, respectively. Material exposure–outcome overlap was therefore not expected in the primary ALM–ALT, ALM–FinnGen T2D, and ALT–FinnGen T2D comparisons. The BMI meta-analysis included UK Biobank participants and could therefore share participants with the ALM GWAS. We therefore treated BMI-adjusted MVMR as a sensitivity analysis and evaluated alternative cross-exposure covariance scenarios using the prespecified ρ grid. T2DGGI comparisons could likewise be affected by overlap with UK Biobank or MVP component cohorts. No quantitative covariance correction for participant overlap was applied because the required covariance information was unavailable.

**Figure 1:**
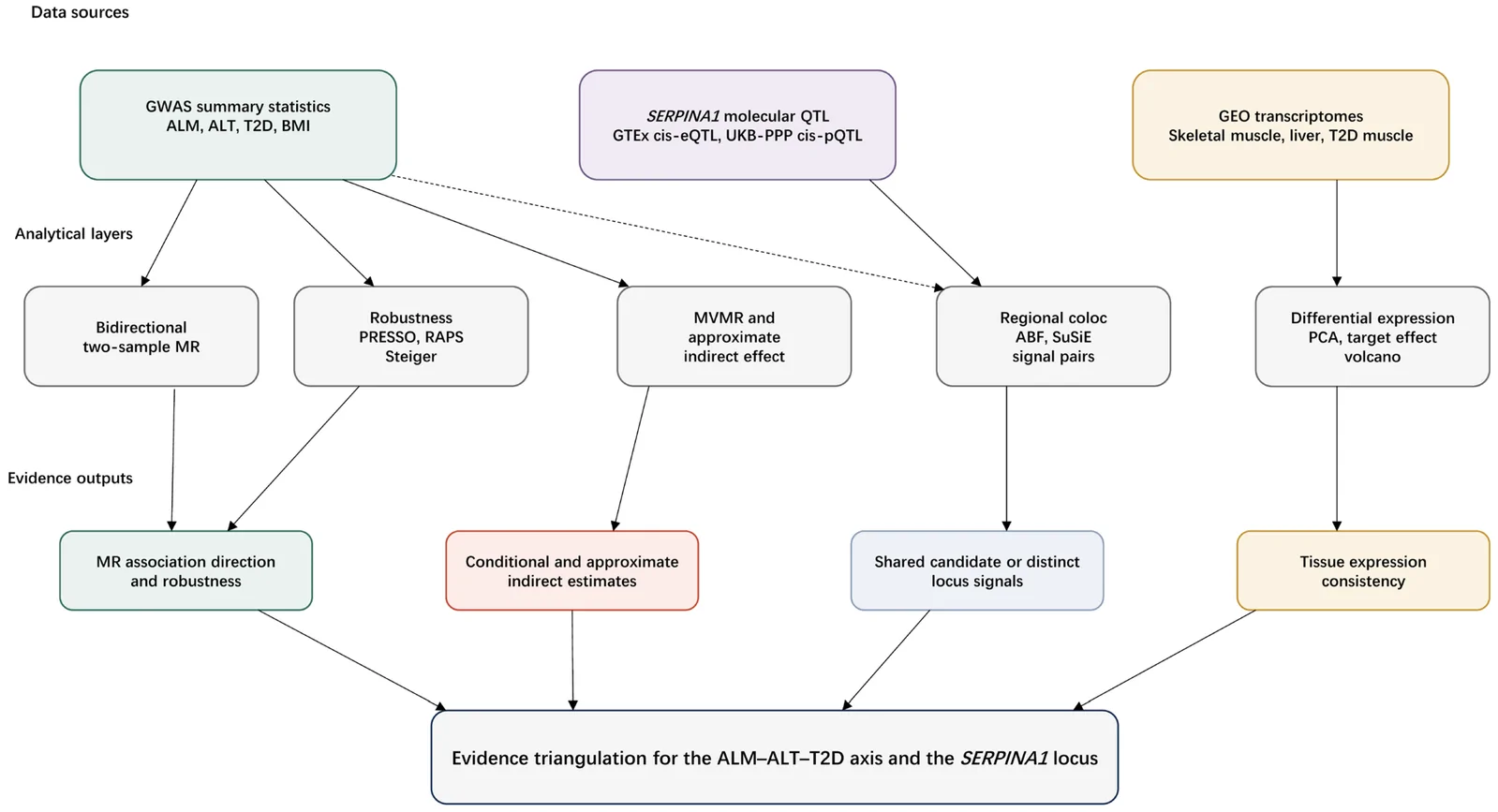
Study design and multilayer evidence-integration framework. The framework integrates GWAS summary statistics for ALM, ALT, T2D, and BMI, *SERPINA1* cis-eQTL/cis-pQTL data, and GEO transcriptomic data. Bidirectional two-sample MR, robustness analyses, MVMR, approximate summary-data indirect-effect analysis, ABF/SuSiE colocalization, and cohort-specific differential expression constitute analytically distinct but complementary evidence layers. Solid arrows indicate the main data-to-analysis and analysis-to-output flow, whereas the dashed arrow indicates the direct use of GWAS summary statistics in regional colocalization. Green boxes denote the GWAS/MR evidence stream; purple and blue boxes denote molecular-QTL and colocalization evidence; gold boxes denote transcriptomic evidence; the salmon box denotes conditional and approximate indirect-effect estimates; gray boxes denote analytical procedures; and the navy-outlined box denotes the final evidence-integration step. The diagram represents the analysis workflow and does not indicate that any layer established a biological mechanism.

Throughout the manuscript, MR and MVMR results were described as MR estimates or MR-based genetic associations. MR estimates have a causal interpretation only if the instrumental-variable assumptions hold. Positive colocalization results were uniformly described as shared candidate signals. Comparisons that did not converge or did not produce an eligible credible set were defined as non-informative for signal-specific colocalization. Estimable comparisons dominated by H3 or with low H4 were described as not supporting a shared signal. An overview of the study design and evidence-integration framework is provided in Figure 1.

For reporting and interpretation, the analyses were organized into primary, sensitivity, and exploratory tiers. The six unfiltered IVW estimates and the prespecified two-exposure ALM + ALT→FinnGen T2D MVMR model were the primary MR analyses. Complementary MR estimators, MR-RAPS, MR-PRESSO, Steiger filtering, one-pass RadialMR and RadialMVMR, robust or BMI-adjusted MVMR, and candidate-region exclusion were treated as sensitivity analyses. The path-specific indirect-effect analysis and the strict same-SNP-set analysis were exploratory. The same-SNP-set analysis was retained only as a weak-instrument diagnostic. Candidate-region colocalization was hypothesis-driven, whereas the molecular-QTL, transcriptomic, GSEA, and STRING analyses were used as supportive or limiting evidence rather than mechanistic validation.

We applied Benjamini–Hochberg false-discovery rate and Bonferroni corrections to the six prespecified unfiltered IVW directions. The Bonferroni threshold for these six tests was 0.05/6 = 0.0083. Adjusted *p*-values were also reported within the relevant method-specific families for sensitivity diagnostics and MVMR estimates. Benjamini–Hochberg correction was used for the transcriptomic, GSEA, and network-permutation analyses as specified in the Supplementary Methods. Empirical *p*-values from the path-specific bootstrap were interpreted only within that exploratory analysis and were not used to infer biological mediation. Colocalization was evaluated using posterior probabilities and prespecified prior-sensitivity analyses rather than frequentist multiple-testing correction.

Table 1 summarizes sample sizes, ancestry, analytical roles, genome builds, and known or potential sample overlap for the main genetic datasets. Detailed data sources and access information are provided in Supplementary Table S1a. Data dictionaries, status definitions, record counts, and figure source-data maps are provided in Supplementary Tables S9a–d.

### 2.2. Genetic Instruments and Bidirectional MR

The six prespecified directions were ALM→ALT, ALT→ALM, ALM→T2D, T2D→ALM, ALT→T2D, and T2D→ALT. Analyses were conducted in R 4.5.1 using TwoSampleMR 0.6.22 [15]. The primary instrument threshold was *p* < 5 × 10^−9^ for ALM and *p* < 5 × 10^−8^ for ALT and T2D. BMI instruments were selected at *p* < 1 × 10^−8^, with *p* < 5 × 10^−8^ used in the sensitivity analysis.

Candidate instruments were restricted to autosomal biallelic SNPs with minor-allele frequency (MAF) ≥ 0.01 and *F* = (*β*/SE)^2^ ≥ 10. LD clumping was performed with PLINK 1.9.0-b.7.11 and the 1000 Genomes European reference panel (r^2^ < 0.001; window, 10,000 kb) [36,37]. Exposure and outcome alleles were harmonized using TwoSampleMR::harmonise_data(action = 2). Palindromic SNPs whose orientation could be determined from allele frequencies were retained, whereas variants with ambiguous alignment or incompatibility were excluded.

The multiplicative random-effects inverse-variance-weighted (IVW) method was the primary estimator. MR-Egger, the weighted median, and the weighted mode were used as complementary estimators. Cochran’s Q assessed heterogeneity, and the MR-Egger intercept assessed directional horizontal pleiotropy. I^2^_GX_ assessed regression-dilution risk in MR-Egger, whereas leave-one-out analysis assessed the influence of individual instruments. The primary conclusions required overall directional concordance across the prespecified estimators. Statistical significance with one estimator alone was insufficient to establish robustness across methods.

MR-RAPS (mr.raps 0.4.3) assessed the influence of weak instruments and dispersed pleiotropy [38]. MR-PRESSO (MRPRESSO 1.0) assessed the influence of outliers on MR estimates [39]. Steiger filtering, one-pass RadialMR (version 1.2.1), and correction for the six MR directions were prespecified sensitivity analyses [40,41]. Complete fitting warnings, outlier rules, estimates before and after filtering, and multiplicity-adjusted results are provided in Supplementary Method S3 and Supplementary Table S2. Sensitivity analyses did not replace the unfiltered primary IVW estimates. Complete software and package versions are provided in Supplementary Table S1b.

### 2.3. Multivariable MR and Exploratory Path-Specific Analysis

We fitted an MVMR model with ALM and ALT as exposures and FinnGen R12 T2D as the outcome. This model estimated the ALT conditional effect and the ALM direct effect. Genome-wide significant ALM and ALT instruments were pooled, jointly LD-clumped, and harmonized. MVMR 0.4.8 was used for the primary MVMR-IVW estimate, exposure-specific conditional *F* statistics, and Q_A heterogeneity testing. MendelianRandomization 0.10.0 was used for cross-software random-effects MVMR sensitivity analysis, and RMVMR 0.4.5 was used for one-pass RadialMVMR sensitivity analysis [42,43,44]. Outlier SNPs were removed in a single prespecified pass; filtering was not repeated to obtain a nonsignificant Q_A. A conditional *F* statistic < 10 was treated as a warning of possible conditional weak-instrument bias.

**Figure 2:**
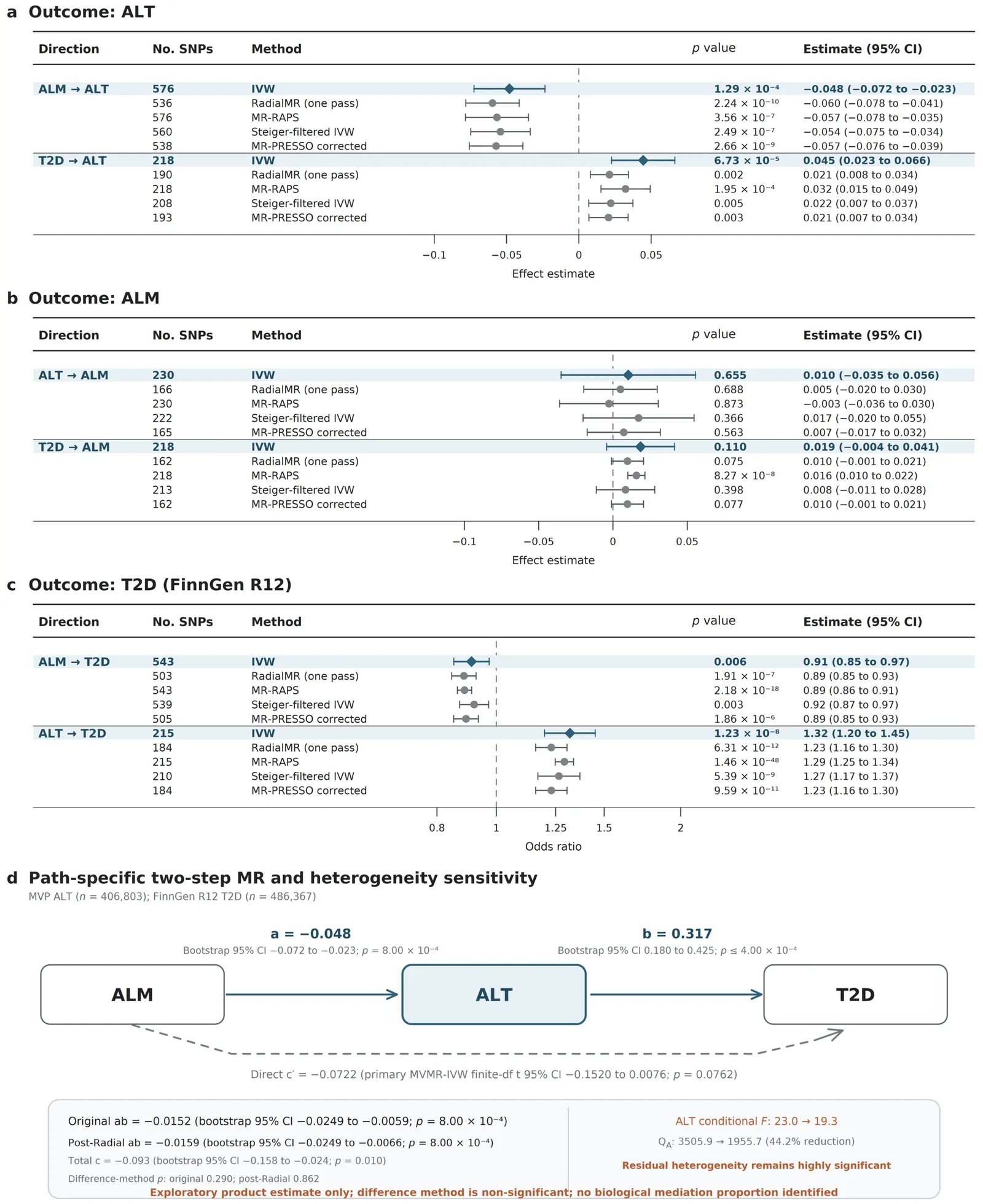
Prespecified-direction MR estimates and exploratory path-specific two-step MR. (**a**–**c**) The six prespecified directions are shown by outcome; points indicate effect estimates and horizontal lines indicate 95% CIs. Blue diamonds and blue-shaded rows denote the primary IVW estimates, whereas gray circles denote the sensitivity estimates. Dashed vertical lines indicate the null value, 0 for β estimates and 1 for ORs. Primary IVW, MR-RAPS, Steiger-filtered IVW, MR-PRESSO, and one-pass RadialMR estimates are shown separately. Continuous outcomes are reported as GWAS *β* coefficients, whereas FinnGen R12 T2D is reported as an OR. (**d**) Path-specific analysis: a = −0.0479, b = 0.3166, c = −0.0933, and c′ = −0.0722. Solid blue arrows denote paths a and b, whereas the dashed gray arrow denotes the direct effect c′. The blue ALT box identifies the intermediate phenotype in the exploratory statistical pathway, and orange text highlights the remaining conditional-instrument-strength and heterogeneity concerns. The original product-based indirect effect was ab = −0.0152 (95% CI, −0.0249 to −0.0059; shared-sampling bootstrap *p* = 0.0008). After one-pass RadialMVMR, the product-based indirect effect was ab = −0.0159 (95% CI, −0.0249 to −0.0066; *p* = 0.0008). The difference-based estimates were −0.0211 (*p* = 0.290) in the original instrument set and 0.0022 (*p* = 0.862) after one-pass RadialMVMR. The ALT conditional *F* statistic decreased from 23.0 to 19.3, while Q_A decreased by 44.2% but remained significant. Because the product- and difference-based estimates were discordant, the panel shows an exploratory statistical pathway only and does not report a mediation proportion or biological mediation conclusion.

The primary MVMR inference rule used a finite-sample t distribution. For models with K jointly clumped instruments and L exposures, we calculated t = *β*/SE with df = K − L. Two-sided *p*-values were obtained from this finite-sample t distribution. The corresponding 95% confidence intervals were calculated as *β* ± t_0.975,df_ × SE. Derived results recorded the inference distribution and the actual degrees of freedom. MendelianRandomization results served only as cross-software sensitivity analyses. They were not used for the primary MVMR estimate, main-text conclusions, or Figure 2.

MVMR-Lasso [45], MVMRcML-DP [46], the ALM + ALT + BMI→T2D model, and one-pass RadialMVMR were treated as sensitivity analyses. The BMI dataset could share UK Biobank participants with the ALM dataset. The BMI-adjusted model therefore served as a conditional sensitivity analysis and did not replace the primary two-exposure model. Complete model specifications, conditional *F* statistics, Q_A statistics, and instrument handling are provided in Supplementary Method S4 and Supplementary Tables S2 and S3.

We performed exploratory path-specific two-step MR to examine the ALM → ALT → T2D candidate pathway [47,48]. Genome-wide significant ALM instruments were used to estimate the ALM→ALT path (a) and the total ALM→T2D effect (c). The jointly clumped ALM + ALT instrument set was used to estimate the conditional ALT→T2D path (b) and the direct ALM→T2D effect (c′). All T2D paths used FinnGen R12, with effect alleles aligned consistently. We simultaneously re-estimated a, b, c, and c′ using 5000 shared SNP–trait-parameter bootstrap replicates. These replicates propagated covariance between paths and yielded product-based (ab) and difference-based (c − c′) decompositions. One-pass RadialMVMR was treated as a sensitivity analysis. Results reaching the empirical two-sided *p*-value resolution limit were reported as *p* ≤ 0.0004.

The product-based indirect effect and its ratio to the total effect were treated only as exploratory statistical decompositions. The strict same-SNP-set analysis used a delete-one-SNP jackknife to estimate path covariance. Because the ALT conditional *F* statistic was only 4.79, this analysis served solely as a weak-instrument diagnostic. Summary-data two-step MR cannot verify sequential ignorability, the full exclusion restriction, exposure–intermediate interaction, or parallel metabolic pathways. Accordingly, no ratio was interpreted as an identified biological mediation proportion.

#### Candidate-Region Exclusion Sensitivity Analysis

To assess dependence on the candidate region, we defined the ±500 kb interval around rs17580 in GRCh37 (chr14:94,347,262–95,347,262). Variants within this interval were removed from the harmonized bidirectional MR datasets and the jointly clumped ALM + ALT MVMR dataset. We then re-estimated the six MR directions and the ALM + ALT→FinnGen T2D MVMR model. Instrument thresholds, clumping criteria, allele harmonization, estimators, and the zero cross-exposure covariance assumption were unchanged. Instruments were not reselected or reclumped after regional exclusion; this analysis was therefore treated only as sensitivity evidence.

### 2.4. Selection of the SERPINA1 Candidate Region and Phenotype Colocalization

We selected *SERPINA1* based on prior regional associations and the functional relevance of protein-altering variants. Genetic evidence linked this region to liver enzymes and liver disease [22]. The biological plausibility of its encoded protein, AAT, in liver and metabolic biology further supported this choice [24]. We did not perform a genome-wide shared-region scan and therefore cannot exclude other shared regions.

All candidate-region inputs were harmonized to GRCh37. For ALT, FinnGen T2D, and eQTL data originating in GRCh38, mapping prioritized rsID and alignment to the 1000 Genomes European reference panel. Records without usable rsIDs were matched by GRCh37 position and unordered allele pairs. The candidate interval was defined as ±500 kb around rs17580, corresponding to GRCh37 chr14:94,347,262–95,347,262. We evaluated locus-level shared-signal hypotheses using coloc::coloc.abf() (coloc 5.2.3) [25]. Comparisons included ALM–ALT and each trait paired with T2D from FinnGen or T2DGGI. The primary priors were P1 = P2 = 1 × 10^−4^ and P12 = 1 × 10^−5^, with sensitivity analysis across P12 = 1 × 10^−6^ to 1 × 10^−4^.

SuSiE-coloc was performed in the same GRCh37 region to evaluate complex LD and multiple association signals [26,49,50]. We estimated a signed LD matrix using the 1000 Genomes Project Phase 3 European reference panel (*n* = 503) [37]. Variant order in the matrix exactly matched that in the summary statistics. Fine-mapping used susieR 0.14.2 (L = 10; maxit = 1000), followed by comparison of eligible credible-set signal pairs using coloc::coloc.susie(). Only models that converged and produced an eligible credible set were retained. The number of iterations, prior variance, and signal count were not increased or altered to force a credible set. No signal-specific conclusion was drawn for comparisons that failed to converge or lacked an eligible credible set. PP.H4 was not set to zero for any such comparison.

Although broadly ancestry-matched, this panel was not derived from the corresponding GWAS cohorts and may not fully represent the LD structure in UK Biobank, MVP, FinnGen, or T2DGGI. In-study LD and a second large independent European LD reference panel were unavailable. Consequently, all signal-specific SuSiE-coloc findings were treated as reference-dependent sensitivity evidence. Differences between the external reference panel and the GWAS cohorts could affect the fitted signal counts, credible-set composition, posterior inclusion probabilities, and PP.H4. The limited kriging-RSS analysis identified possible local allele-coding errors or outlying signals [50]. This diagnostic analysis could not correct broader LD mismatch between the reference panel and GWAS cohorts. ABF results were interpreted at the locus level, whereas SuSiE-coloc results were interpreted at the signal level. Potential component-cohort overlap in T2DGGI was evaluated separately. A high PP.H4 indicated compatibility with a shared candidate signal under the specified data, LD reference, and priors. It did not establish a unique causal variant, gene-level causal assignment, or molecular mediation.

### 2.5. SERPINA1 Molecular-QTL Colocalization

Molecular-QTLs related to *SERPINA1* regulation or protein variation were evaluated in the same GRCh37 candidate region. We used eQTL Catalog-harmonized GTEx v8 cis-eQTLs from liver (*n* = 208), skeletal muscle (*n* = 702), and whole blood (*n* = 670) [51,52]. Plasma AAT cis-pQTLs came from the UKB-PPP European discovery sample (*n* = 33,348) [53]. cis-eQTLs were extracted for ENSG00000197249. UKB-PPP pQTLs were restricted to additive models, biallelic SNPs, INFO ≥ 0.80, and MAF ≥ 0.01.

All molecular-QTL–trait combinations underwent locus-level ABF analysis. Signal-specific SuSiE-coloc results were reported only when models for both datasets converged, and each produced an eligible credible set. Other comparisons were classified as non-informative for signal-specific colocalization. These comparisons were neither assigned PP.H4 = 0 nor interpreted as evidence against a shared signal. Complete results for these comparisons are provided in Supplementary Table S5. Because UKB-PPP and T2DGGI may both contain UK Biobank components, signal-specific pQTL–T2DGGI results require cautious interpretation.

### 2.6. Supportive External-Omics Analyses

We analyzed five prespecified comparisons across three GEO datasets using established differential-expression and annotation workflows [54,55,56,57,58,59]. We also performed preranked GSEA [60,61,62,63] and STRING network analysis [64]. External-omics results served only to support or limit biological interpretation. They were not used to assign a gene-level causal role, identify a molecular mediator, or infer a mechanism. Data preprocessing, differential-expression models, GSEA and leading-edge analyses, and STRING network, centrality, and permutation parameters are provided in Supplementary Methods S9–S11.

## 3. Results

### 3.1. Bidirectional Genetic Associations Among ALM, ALT, and T2D

The six prespecified MR directions included 215–576 instruments per direction. Minimum *F* statistics ranged from 29.70 to 32.49, all above 10 (Supplementary Table S2). Genetically predicted higher ALM was associated with lower ALT (576 SNPs; *β* = −0.0479, 95% CI, −0.0725 to −0.0234, *p* = 1.29 × 10^−4^). Higher genetically predicted ALM was also associated with lower T2D risk (543 SNPs; OR = 0.911, 95% CI, 0.852 to 0.974, *p* = 0.00595). Genetically predicted higher ALT was associated with higher T2D risk (215 SNPs; OR = 1.319, 95% CI, 1.199 to 1.450, *p* = 1.23 × 10^−8^). All three directions passed the six-direction Bonferroni threshold of 0.0083.

ALT→ALM was not significant (230 SNPs; *β* = 0.0103, 95% CI, −0.0349 to 0.0555, *p* = 0.655), nor was T2D→ALM (218 SNPs; *β* = 0.0186, 95% CI, −0.0042 to 0.0414, *p* = 0.110). The IVW estimate for T2D→ALT was statistically significant (218 SNPs; *β* = 0.0445, 95% CI, 0.0226 to 0.0664, *p* = 6.73 × 10^−5^). However, the weighted median and MR-Egger estimates were not significant, and the weighted-mode estimate was in the opposite direction. Thus, ALM→ALT, ALM→T2D, and ALT→T2D were directionally concordant overall across the prespecified estimators, whereas T2D→ALT was classified as a method-dependent exploratory result.

Sensitivity diagnostics indicated substantial residual heterogeneity in multiple directions. In the original instrument sets, the ALT→T2D MR-Egger intercept was significant after within-group BH adjustment (intercept = 0.00768, within-group BH-FDR *p* = 0.0134). The T2D→ALM intercept also passed this adjustment (intercept = 0.00369, within-group BH-FDR *p* = 0.0358). The ALM→ALT and ALM→T2D intercepts reached only nominal significance (BH-FDR *p* = 0.0563). After one-pass RadialMR, no MR-Egger intercept remained significant after BH adjustment. Outlier handling reduced some diagnostic signals but did not rule out directional horizontal pleiotropy or residual heterogeneity. The MR/MVMR estimates therefore represent genetic associations, not established unbiased causal effects. The direction-specific MR estimates and sensitivity analyses are summarized in Figure 2a–c.

### 3.2. Sensitivity Analyses and Residual Heterogeneity

After Steiger filtering, ALM→ALT, ALM→T2D, and ALT→T2D retained their directions and statistical support. ALT→ALM and T2D→ALM remained nonsignificant, whereas T2D→ALT remained inconsistent across estimators. MR-RAPS generally retained the directions of these three associations, but some over-dispersion fits produced fallback warnings. These estimates were therefore reported as sensitivity results with their fitting warnings. They did not establish that heterogeneity or pleiotropy had been excluded.

One-pass RadialMR and MR-PRESSO reduced some heterogeneity and identified overlapping outlier SNPs, but Q tests remained significant after filtering. The primary directions of ALM→ALT, ALM→T2D, and ALT→T2D were unchanged. Complete effect estimates, warnings, outlier rules, and multiplicity-adjusted results are provided in Supplementary Method S3 and Supplementary Table S2. Filtered results did not replace the unfiltered primary IVW estimates.

### 3.3. Conditional Effects and Exploratory Statistical Decomposition of the ALM–ALT–T2D Pathway

The primary ALM + ALT→FinnGen T2D MVMR included 462 harmonized instruments and two exposures. Conditional *F* statistics were 100.74 for ALM and 22.96 for ALT. Finite-sample t-based inference used df = 462 − 2 = 460. *p*-values and 95% CIs were calculated from the same t_460_ distribution. The ALT conditional effect was positive (*β* = 0.3166, 95% CI, 0.1936 to 0.4396, OR = 1.372, *p* = 6.14 × 10^−7^). The ALM direct effect was negative but not statistically significant (*β* = −0.0722, 95% CI, −0.1520 to 0.0076, OR = 0.930, *p* = 0.0762). After one-pass RadialMVMR removed 12 SNPs, the ALT conditional effect remained positive (*β* = 0.3310, 95% CI, 0.2287 to 0.4334, *p* = 5.65 × 10^−10^). The ALM direct effect was *β* = −0.0955 (95% CI, −0.1577 to −0.0332, *p* = 0.0028).

In 5000 shared-sampling bootstrap replicates, the ALM→ALT path had *p* = 0.0008, and the ALT conditional→T2D path reached the empirical two-sided resolution limit (*p* ≤ 0.0004). The product-based indirect effect had an empirical *p*-value of 0.0008. The difference-based estimate was not significant in the original instrument set (*p* = 0.290) or after one-pass RadialMVMR (*p* = 0.862). One-pass RadialMVMR reduced Q_A from 3505.88 to 1955.70, a 44.2% reduction, but residual heterogeneity remained significant. The bootstrap propagated uncertainty in path parameters at the summary-statistic level. It could not verify sample-overlap covariance or establish biological mediation. The exploratory path-specific decomposition is shown in Figure 2d.

MVMR-Lasso and MVMRcML-DP both retained a positive ALT conditional effect. The ALM direct effect was negative in invalid-instrument robust analyses, but its magnitude depended on the model and instrument selection. In the BMI-adjusted model, the ALT and BMI directions were stable, whereas the ALM effect ranged from nonsignificant to borderline or significant. The strict same-SNP-set analysis yielded an ALT conditional *F* statistic of only 4.79, indicating insufficient conditional instrument strength. It therefore served only as a weak-instrument diagnostic, not as support for mediation or a basis for calculating a mediation proportion.

#### Sensitivity Analyses After Exclusion of the *SERPINA1* Candidate Region

Complete instrument counts, MR and MVMR estimates, heterogeneity statistics, and conditional instrument strength after regional exclusion are provided in Supplementary File S1. Excluding the candidate interval did not change the directions of ALM→ALT, ALM→T2D, or the conditional ALT→T2D effect. However, the ALT→T2D effect increased, and the ALT→ALM estimate moved towards the null. These results suggest that the primary associations were not entirely driven by the candidate region. Nevertheless, variants in this region influenced some ALT-related analyses.

### 3.4. Single- and Multi-Signal Colocalization at the SERPINA1 Region

Within the harmonized GRCh37 candidate region, locus-level ABF supported a shared signal for several phenotype pairs. It could not distinguish individual signals or resolve uncertainties arising from potential cohort overlap or external-LD differences in signal-specific analyses. The primary locus-level conclusions were stable when P12 varied across the prespecified range of 1 × 10^−6^ to 1 × 10^−4^.

**Figure 3:**
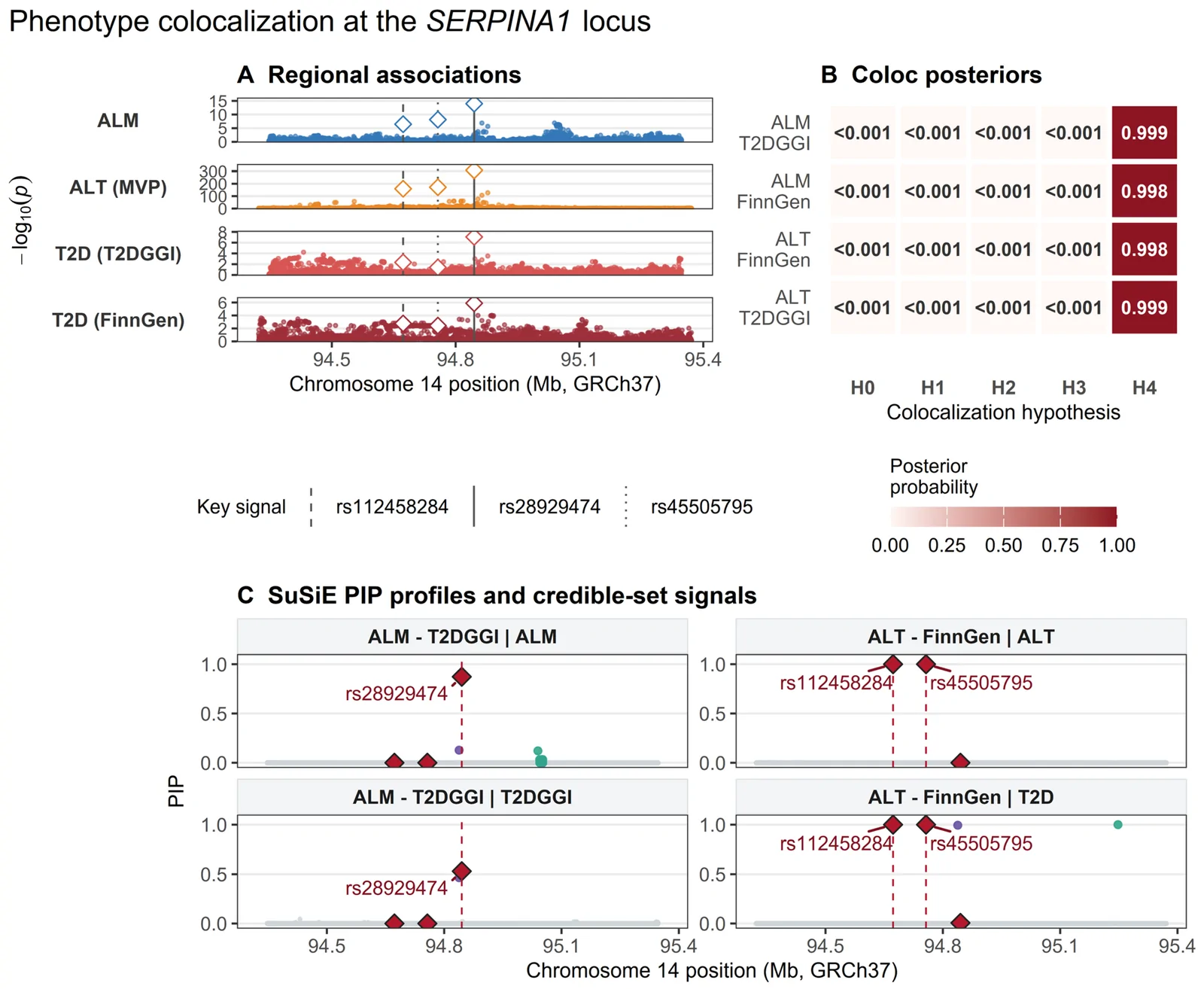
Phenotype colocalization in the GRCh37 *SERPINA1* region. (**A**) Regional association plots for ALM, MVP ALT, T2DGGI, and FinnGen T2D; all key reference signals were harmonized to GRCh37. Point colors distinguish the four datasets. Open diamonds mark the three reference variants, and the dashed, solid, and dotted vertical lines denote rs112458284, rs28929474, and rs45505795, respectively, as defined in the key. (**B**) Locus-level coloc/ABF posterior probabilities for ALM/ALT with the two T2D datasets; locus-level H4 denotes regional compatibility with a shared signal and is not equivalent to signal-specific fine-mapping. Cell values report the posterior probabilities for hypotheses H0–H4, with darker red indicating a higher posterior probability. (**C**) SuSiE PIPs and credible sets are shown only for comparisons that converged and produced eligible credible sets. Gray points represent background variants, colored points indicate SuSiE signal components, and red diamonds and dashed vertical lines mark the labeled reference variants. Panel-strip labels identify the phenotype pair and the dataset for which PIPs are displayed. The rs28929474-related ALM–T2DGGI signal may be affected by component-cohort overlap. ALT–FinnGen supported signal components related to rs112458284 and rs45505795. This result depended on an external LD reference panel and did not independently corroborate rs28929474. ALM–FinnGen was non-informative because the FinnGen T2D model in this comparison did not produce an eligible credible set. PP.H4 was not set to zero for this non-informative comparison. External LD and sample overlap limit interpretation of PIPs and PP.H4; a high PP.H4 cannot determine a unique causal variant, gene-level causal assignment, or molecular mediator.

Signal-specific SuSiE results depended on the dataset and comparison. ALM–T2DGGI showed PP.H4 = 0.99903 for an rs28929474-related signal pair. Potential component-cohort overlap and reliance on an external LD reference limited interpretation of this result. It therefore supported a candidate signal, not independent replication or definitive fine-mapping. ALT–FinnGen T2D produced eligible credible sets and provided signal-specific support for components related to rs112458284 and rs45505795. ALM–FinnGen was non-informative for signal-specific colocalization because the FinnGen T2D model in this comparison did not produce an eligible credible set. Separately, the ALT–T2DGGI comparison failed to converge and was also non-informative. Thus, FinnGen supported other ALT-related signals but did not independently replicate rs28929474. Regional association plots, locus-level ABF results, and signal-specific SuSiE results are shown in Figure 3A–C; complete phenotype-colocalization results are provided in Supplementary Tables S4a–S4c.

### 3.5. Molecular-QTL Colocalization

**Figure 4:**
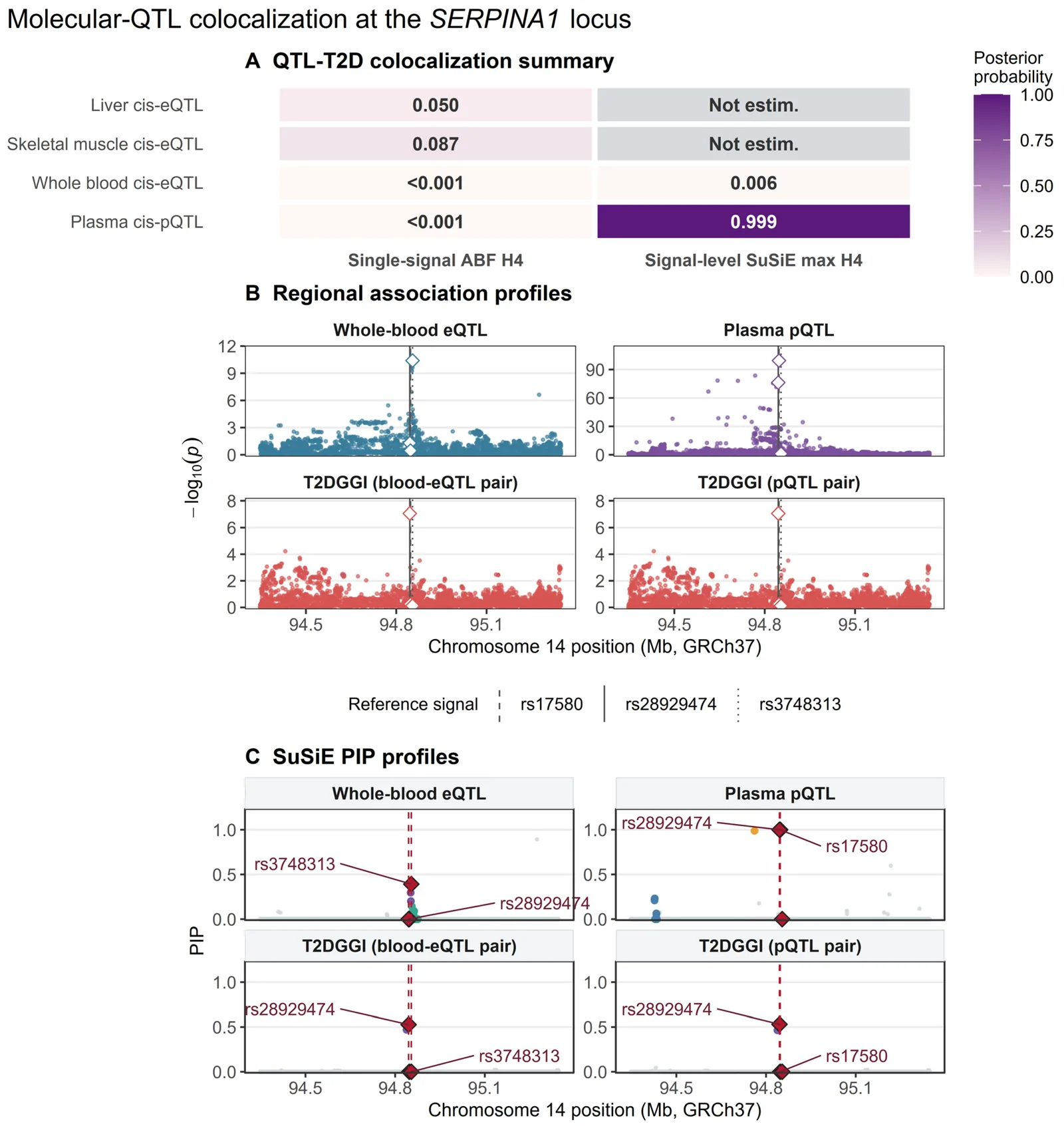
Key molecular-QTL–T2D colocalization results in the GRCh37 *SERPINA1* region. (**A**) Locus-level ABF H4 for liver, skeletal-muscle, and whole-blood cis-eQTLs and plasma cis-pQTLs with T2DGGI, together with the maximum SuSiE H4 among eligible signal pairs. Cell values report posterior probabilities, with darker purple indicating a higher probability. Gray “Not estim.” cells denote non-informative signal-specific comparisons for which no eligible credible set was produced; they do not indicate PP.H4 = 0 or evidence against a shared signal. (**B**) Representative regional associations for whole-blood eQTLs and plasma pQTLs. Blue, purple, and red points distinguish the molecular-QTL and T2DGGI datasets. Open diamonds mark the reference variants, and the dashed, solid, and dotted vertical lines denote rs17580, rs28929474, and rs3748313, respectively, as defined in the key. (**C**) SuSiE PIPs show separation between the major whole-blood eQTL and T2DGGI signals. Gray points represent background-variant PIPs, colored points indicate SuSiE signal components, and red diamonds with dashed vertical lines mark the labeled reference variants. Leader lines connect variant labels to their corresponding points. Plasma pQTLs and T2DGGI showed support for a shared signal only in an rs28929474-related component. The pQTL–T2DGGI result may be affected by component-cohort overlap. pQTL effects may also reflect protein abundance, structure, secretion, or assay-binding properties. This result therefore provides supportive evidence, not independent protein-level corroboration. Signal-specific results depend on the external 1000 Genomes EUR LD reference panel. Neither PIPs nor PP.H4 alone can establish a unique causal variant.

Complete locus-level and signal-specific molecular-QTL results are provided in Supplementary Table S5. Signal-specific analyses of liver, skeletal-muscle, and whole-blood eQTLs provided no consistent evidence of shared signals. Plasma pQTLs and T2DGGI showed high signal-specific H4 for an rs28929474-related component. Potential UKB-PPP–T2DGGI sample overlap limited interpretation as independent corroboration, and the result did not establish protein-level mediation. Molecular-QTL results were therefore treated only as supportive and limiting evidence for the candidate region. Key molecular-QTL–T2D colocalization comparisons are shown in Figure 4A–C.

### 3.6. External Omics Did Not Provide Consistent Mechanistic Support

External transcriptomics, preranked GSEA, and STRING network analyses did not produce a consistent candidate-region signal across tissues and cohorts (Supplementary Tables S6–S8 and Supplementary Figures S1 and S2). These negative or inconsistent results limited the biological interpretation of the colocalization findings.

## 4. Discussion

### 4.1. Principal Findings and Overall Interpretation

This study integrated evidence from bidirectional MR, MVMR, exploratory pathway analysis, and candidate-region colocalization. Analyses of European-ancestry summary statistics linked genetically predicted higher ALM to lower ALT and lower T2D risk. Genetically predicted higher ALT was associated with higher T2D risk, but reverse T2D→ALT estimates were method-dependent. In MVMR, the ALT conditional effect was relatively stable, whereas the ALM direct effect depended on the estimator and outlier handling. The decomposition was compatible with an ALT-related statistical pathway, but product- and difference-based estimates were discordant and residual heterogeneity remained. Regional analyses distinguished the rs28929474-related ALM–T2DGGI signal from other ALT-related signals supported by FinnGen. Interpretation of the ALM–T2DGGI result remained limited by potential cohort overlap.

ALM represents muscle mass and cannot substitute for clinical sarcopenia. MVP ALT beta coefficients are expressed on an inverse-normal-transformed GWAS scale. Candidate-region evidence is further limited by reliance on external LD and potential sample overlap. The study therefore supports directionally connected MR-based genetic associations and multiple competing regional explanations rather than a validated biological pathway.

### 4.2. Directional Relationships and Statistical Indirect Effects

ALM represents a genetic phenotype related to appendicular lean mass and cannot be equated with clinically defined sarcopenia [4]. Skeletal muscle contributes to insulin-stimulated glucose uptake, glycogen storage, and energy utilization. Reduced muscle mass may be associated with impaired glucose disposal, insulin resistance, and ectopic lipid deposition [5,6,65]. The observed association of genetically predicted higher ALM with lower T2D risk was broadly compatible with this metabolic framework [28]. However, ALM does not capture muscle strength, muscle quality, intramuscular fat infiltration, or physical performance [29]. The present findings therefore support an association between muscle-mass-related genetic architecture and T2D risk, not an established causal effect of sarcopenia itself on T2D.

Previous MR studies have separately examined relationships between liver enzymes and body composition and statistical mediation involving sarcopenia-related traits, cardiometabolic disease, and insulin resistance [30,66]. These studies support the biological and epidemiological relevance of the question. However, their exposure definitions, instruments, outcome datasets, and mediation-identification assumptions differ from ours. They therefore do not independently replicate the pathway estimates reported here. ALT is also not a single liver-specific molecular exposure; it may reflect hepatocellular injury, hepatic lipid burden, and broader metabolic stress [18,67]. The liver contributes to gluconeogenesis, glycogen metabolism, lipid handling, and insulin clearance [16,17,68]. Higher ALT commonly accompanies obesity-related metabolic dysfunction, insulin resistance, and metabolic dysfunction-associated steatotic liver disease and is associated with higher T2D risk [18,67]. Because body-composition and T2D associations may vary by sex and ancestry [21], the present interpretation should remain limited to the analyzed ALM phenotype, MVP ALT, and European-ancestry T2D datasets.

The directional results were asymmetric. ALM→ALT and ALT→T2D retained broadly consistent directions across estimators and outlier-sensitive analyses, and the total ALM→T2D effect was also supported. In contrast, T2D→ALT estimates were inconsistent across estimators and decreased in magnitude by 52.4% after Radial filtering. T2D→ALT should therefore be treated as a method-dependent exploratory result and cannot support a stable bidirectional causal relationship between ALT and T2D. Directionally connected pairwise associations do not by themselves establish a causal pathway. Consistency across robust estimators indicates relative stability under the current instruments and models. It does not validate the exclusion restriction or rule out horizontal pleiotropy.

In MVMR with FinnGen T2D, the ALT conditional effect remained positive across primary IVW, one-pass RadialMVMR, MVMR-Lasso, and MVMRcML-DP analyses. The ALM direct effect was nonsignificant in the primary model and varied across sensitivity models. This conditional association alone does not establish ALT as a biological mediator between ALM and T2D. Residual heterogeneity and model dependence of the ALM effect leave open explanations involving horizontal pleiotropy, signal heterogeneity, or parallel metabolic pathways. These pathways may involve insulin resistance, adiposity, hepatic steatosis, or other metabolic processes. The present summary-data design could not directly identify or exclude these alternatives [18,30,67].

Although the product-based indirect-effect estimate was nonzero, its discordance with the difference-based estimate precluded identifying ALT as a biological mediator between ALM and T2D. One-pass RadialMVMR reduced Q_A by 44.2%, but significant residual heterogeneity remained, indicating that outlier removal did not resolve this concern. The 5000 shared-sampling parameter bootstrap replicates propagated covariance among path estimates as represented in the summary-data simulation. This procedure could not verify sequential ignorability or the exclusion restriction, assess exposure–intermediate interactions, or exclude parallel metabolic pathways [47,48]. These findings therefore support only an exploratory ALT-related statistical pathway, and no biological mediation proportion was reported.

### 4.3. Multi-Signal Architecture and Region-Specific Effects at SERPINA1

The *SERPINA1* findings highlight the complexity of shared genetic signals across traits. Locus-level ABF produced high H4 for several comparisons of ALM/ALT with FinnGen or T2DGGI T2D, whereas signal-specific SuSiE results depended on the dataset and comparison. ALM–T2DGGI yielded PP.H4 = 0.99903 for an rs28929474-related signal pair, but this comparison may be affected by component-cohort overlap. ALT–FinnGen provided signal-specific cross-cohort support for components related to rs112458284 and rs45505795. By contrast, ALM–FinnGen did not produce an eligible T2D credible set, and ALT–T2DGGI did not converge. Thus, the cross-cohort FinnGen evidence pointed to a different set of ALT-related signals rather than independently replicating rs28929474. Given the external LD reference, the multi-signal regional architecture, and the relatively low frequency of rs28929474, high PIPs or PP.H4 values cannot be equated directly with robust fine-mapping or a unique causal variant.

Differences between locus-level ABF and SuSiE-coloc reflect different analytical levels and assumptions and should not be treated as simple contradictions. Locus-level pQTL colocalization was dominated by PP.H3, suggesting distinct causal signals within the region. After signal decomposition, the rs28929474-related pQTL component showed strong support for sharing with T2DGGI. However, UKB-PPP and T2DGGI may contain overlapping UK Biobank components, so this result cannot be considered a separate validation. An independent protein platform and T2D data excluding the relevant UK Biobank components were unavailable. The current evidence cannot confirm changes in circulating AAT abundance or establish protein-level mediation among ALM, ALT, and T2D.

After alignment to the target allele T, rs28929474-T was associated with higher ALM (*β* = 0.0527), higher ALT (*β* = 0.3423), and lower FinnGen and T2DGGI T2D risk. However, the local ALT direction at this variant was discordant with the genome-wide average ALM→ALT direction, and FinnGen and T2DGGI did not fully point to the same signal. Associations of the same allele with multiple phenotypes do not confirm a linear ALM→ALT→T2D pathway. Allelic heterogeneity, multiple LD-related variants, or horizontal pleiotropy may explain why local effects differ from genome-wide average effects.

The candidate region remains biologically plausible, but biological plausibility is not evidence for gene-level causal assignment. *SERPINA1* encodes AAT, whose misfolding, hepatocellular retention, and reduced circulating levels are linked to Z-allele-associated liver phenotypes [69]. Population studies have also associated heterozygous PiZ carriage with elevated liver enzymes and liver-cirrhosis risk [22,70]. Animal and cellular models suggest that exogenous AAT can improve glucose tolerance and beta-cell function in the context of islet amyloid [24]. It may also reduce beta-cell apoptosis under inflammatory conditions. Another study reported that AAT enters beta cells through clathrin-mediated endocytosis and affects JNK activation, mitochondrial dysfunction, and caspase-9-related apoptotic signaling [71]. These findings increase the biological plausibility of the *SERPINA1* region but do not directly explain the present pQTL effects or establish treatment effects in patients with T2D. Experimental AAT treatment and a lifelong genetically influenced plasma-protein measurement are not equivalent exposures. Plasma measurements may also reflect protein folding, secretion, intracellular retention, and assay-binding characteristics. The direction of a plasma pQTL should therefore not be equated simply with the amount of biologically active AAT.

Molecular-QTL and external-omics results further constrain the mechanistic interpretation. Under the prespecified rules, liver, skeletal-muscle, and whole-blood eQTLs provided no consistent signal-specific support for shared signals. The major whole-blood eQTL signals were separated from the shared components of ALM, ALT, and T2D. Plasma pQTLs provided more proximal protein-level shared support in some comparisons, but protein structural changes, secretion, assay binding, and regulation of neighboring genes cannot be excluded. In parallel, external transcriptomics, preranked GSEA, and STRING network analyses did not produce consistent *SERPINA1* mechanism evidence across tissues and cohorts. These negative or inconsistent results limit the biological interpretation of colocalization findings but do not rule out the candidate region.

After regional exclusion, the primary ALM→ALT, ALM→T2D, and conditional ALT→T2D results did not disappear, suggesting that the genome-wide associations were not driven entirely by the *SERPINA1* region. However, the ALT→T2D effect increased, and ALT→ALM approached the null, indicating that regional variants may influence some ALT-related analyses. Three explanations remain compatible with the data: an ALT-related statistical pathway linking ALM and T2D, regional horizontal pleiotropy, and protein-structural or assay-related effects influencing pQTL interpretation. The data cannot distinguish among these explanations or establish *SERPINA1* gene causality or its role as an intervention target.

### 4.4. Study Strengths, Limitations, and Future Directions

This study has four methodological strengths. First, data-source roles were clearly defined. FinnGen R12 provided the MR outcome, MVP provided external ALT data, and T2DGGI was used only for regional colocalization. Second, primary estimates were separated from one-pass outlier sensitivity analyses and cross-software MVMR sensitivity analyses. Third, the analysis reported finite-sample MVMR inference, conditional *F* statistics, Q_A, invalid-instrument robust methods, and shared-sampling bootstrap. Fourth, non-converged models, signal-specific non-informative results, and negative external-omics findings were retained and annotated rather than converted into mechanistic claims.

A major limitation is that the principal GWAS datasets included only or predominantly European-ancestry participants. These datasets comprised UK Biobank ALM, MVP European American ALT, FinnGen T2D, and T2DGGI EUR. Effect estimates, instrument performance, and regional colocalization results may not generalize to other populations. Allele frequencies, LD patterns, body composition, environmental exposures, and T2D susceptibility may differ across populations. This concern is particularly relevant to signal-specific colocalization because variant frequencies and LD patterns may differ substantially across ancestries. For example, a recent MR study found that genetically predicted lean and fat mass showed different relationships with T2D in Western and East Asian populations [21]. We did not analyze East Asian, African, South Asian, or admixed populations. Our study therefore cannot determine whether these associations occur in those populations. Beyond these GWAS-based considerations, case–control studies in North Indian populations have reported population-specific associations of ABCA1/LIPC and INPPL1 polymorphisms with T2D susceptibility [72,73]. These candidate-gene findings further illustrate possible ancestry- and population-context dependence but do not substitute for large ancestry-diverse GWAS or MR replication.

In addition, ALM represents only the muscle-mass dimension and cannot substitute for clinically defined sarcopenia. Both univariable Q and MVMR Q_A indicated residual heterogeneity, and directional consistency across robust methods cannot exclude horizontal pleiotropy. T2D→ALT and the ALM direct MVMR effect were model-dependent. Summary-data pathway analysis cannot verify the full assumptions required for biological mediation. MVP ALT beta coefficients cannot be converted to clinical U/L effects.

Signal-specific SuSiE colocalization relied on an external 1000 Genomes European LD reference panel, whose LD structure may not fully match that of each GWAS cohort. Such mismatch may affect signal separation, variant posterior inclusion probabilities, and credible-set membership, size, and coverage. It may also cause colocalization posterior probabilities to overstate or understate support for a shared signal. LD mismatch may also contribute to non-convergence or failure to obtain an eligible credible set. These outcomes should not be interpreted directly as evidence against a shared signal. Allele alignment, credible-set quality checks, and prior-sensitivity analyses cannot eliminate this limitation. No alternative-LD analysis was performed because in-study LD and an alternative large European LD reference panel were unavailable. Signal-specific findings therefore remain reference-dependent candidate-signal evidence, not definitive fine-mapping or causal assignment. T2DGGI and UKB-PPP may contain UK Biobank components, further limiting interpretation of the relevant comparisons.

Future work should prioritize ancestry-diverse replication of pairwise MR and MVMR analyses using independent T2D datasets without the relevant UK Biobank components. Conditional fine-mapping should use ancestry- and cohort-matched LD. Orthogonal protein platforms could help distinguish genuine changes in AAT abundance from protein-structural or assay-binding effects. Isogenic liver-cell, skeletal-muscle-cell, or liver–muscle co-culture models could then test the effects of key variants on protein folding, secretion, intracellular retention, and metabolic signaling. Until such validation is available, *SERPINA1* should be regarded as a multi-signal candidate region with potential pleiotropy rather than an established gene-level causal assignment or intervention target.

## 5. Conclusions

This study integrated evidence on genetic associations among ALM, ALT, and T2D. Genetically predicted higher ALM was associated with lower ALT and lower T2D risk. Genetically predicted higher ALT was associated with higher T2D risk, but reverse T2D→ALT estimates were method-dependent. MVMR and shared-sampling path decomposition were compatible with an ALT-related statistical pathway. However, residual heterogeneity and disagreement between product- and difference-based estimates prevented identification of biological mediation or a biological mediation proportion.

Locus-level and signal-specific analyses of the *SERPINA1* region revealed dataset-dependent shared candidate signals. Cross-cohort FinnGen analyses supported ALT signal components related to rs112458284 and rs45505795. The current evidence cannot determine a unique causal variant, gene-level causal assignment, longitudinal mediation chain, or molecular mechanism. The primary GWAS datasets predominantly represented European-ancestry populations, and signal-specific colocalization relied on an external European LD reference panel. Generalizability of these findings to other ancestry groups therefore remains uncertain. Independent cohorts, matched LD, and orthogonal functional validation will be needed to evaluate whether this region has mechanistic or intervention relevance.

## Figures and Tables

**Table 1:** Main genetic datasets and potential sample overlap.

Dataset/Trait	Sample Size	Ancestry	Analytical Role	Source Genome Build ^1^	Overlap ^2^
UK Biobank/ALM	450,243	European	MR, MVMR, colocalization	GRCh37	A, B
MVP/ALT	406,803	European American	MR, MVMR, colocalization	GRCh38	B
FinnGen R12/T2D	82,878/403,489 ^3^	Finnish/European	MR, MVMR, colocalization	GRCh38	C
GIANT + UK Biobank/BMI	681,275	European	MVMR	GRCh37	A
T2DGGI/T2D	242,283/1,569,734 ^3^	European	Supplementary colocalization	GRCh37	B
UKB-PPP/*SERPINA1* pQTL	33,348	European	Colocalization	GRCh38 ^4^	A, B
GTEx v8/*SERPINA1* eQTL	Liver, 208; skeletal muscle, 702; whole blood, 670	Multiple ancestries ^5^	Colocalization	GRCh38	C, D

**Table notes:** ^1^ Colocalization analyses used harmonized GRCh37 coordinates and the 1000 Genomes Phase 3 European LD reference panel (*n* = 503). ^2^ A: Potential overlap among UK Biobank-derived datasets. B: Potential component-cohort overlap between T2DGGI and UK Biobank- or MVP-derived datasets. C: No known direct cohort overlap identified with the GWAS datasets compared. D: GTEx tissues may share donors. Exact overlap counts were unavailable; C does not establish zero overlap. ^3^ Cases/controls. ^4^ GRCh37 coordinates embedded in the source variant identifiers were used; 33,348 is the sample size of the *SERPINA1* pQTL data analyzed. ^5^ The overall GTEx cohort was predominantly European American; tissue-specific ancestry proportions were not established. MR, Mendelian randomization; MVMR, multivariable Mendelian randomization. Detailed sources and dataset identifiers are provided in Supplementary Table S1a.

## Data Availability

The GWAS, molecular-QTL, and transcriptomic data used in this study were obtained from public databases or the original data providers. Dataset identifiers, access links, and analysis roles are listed in Supplementary Table S1a. Supplementary File S1 provides the primary analysis scripts, run instructions, input/output descriptions, software-environment information, unrestricted derived results, and plotting data. GWAS, QTL, GEO, LD-reference, MSigDB, and STRING data subject to third-party access or redistribution terms are not redistributed; researchers may obtain the corresponding data from the sources listed in Supplementary Table S1a.

## Supplementary Materials

The following supporting information can be downloaded at: https://www.mdpi.com/article/10.3390/genes17091154/s1, Supplementary Methods S1–S12 [47,48,50,54,55,56,57,58,59,60,61,62,63,64,74]; Supplementary Tables S1a–S1d: data sources, software versions, and sample-overlap assessments; Supplementary Tables S2a–S2e: pairwise MR estimates and sensitivity diagnostics; Supplementary Tables S3a–S3d: multivariable MR, indirect-effect, heterogeneity, and invalid-instrument sensitivity analyses; Supplementary Tables S4a–S4c: phenotype colocalization analyses; Supplementary Tables S5a–S5d: molecular-QTL colocalization analyses; Supplementary Tables S6a–S6c: transcriptomic analyses; Supplementary Tables S7a–S7e: GSEA and cross-context pathway analyses; Supplementary Tables S8a–S8c: STRING network analyses; Supplementary Tables S9a–S9d: data dictionaries, status definitions, record counts, and figure source-data maps; Figure S1: External transcriptomic, pathway-enrichment, and cross-context concordance analyses; Figure S2: STRING network position and permutation analyses of *SERPINA1*; Supplementary File S1: Code and Reproducibility Package.
