# Supplementary material for: Genetic Associations Among Appendicular Lean Mass, Alanine Aminotransferase, and Type 2 Diabetes: Multi-Dataset Mendelian Randomization and Colocalization at the SERPINA1 Candidate Locus
Source: Genes (Basel). 2026 Sep 20;17(9):1154. doi: 10.3390/genes17091154 (PMC13606714; doi:10.3390/genes17091154)
Supplement: Supplementary file 1 [file genes-17-01154-s001.zip › Supplementary_Figures_S1 and S2.pdf]

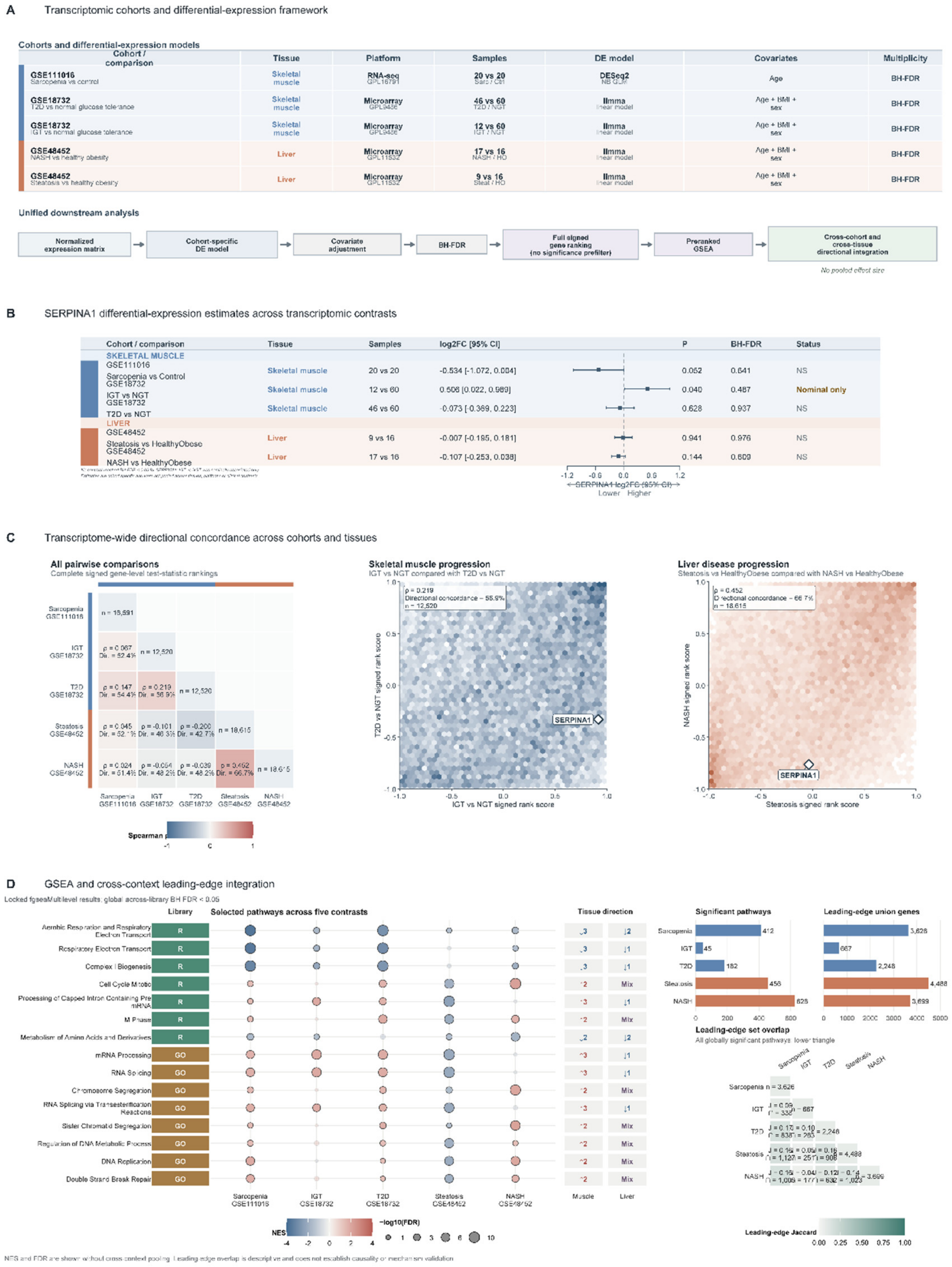

Supplementary Figure S1. External transcriptomic, pathway-enrichment, and cross-context concordance analyses.

(A) Overview of the three GEO datasets, five prespecified contrasts, sample sizes, tissues, and differential-expression models. (B) SERPINA1 differential-expression estimates across the five prespecified contrasts, including effect estimates, 95% confidence intervals, nominal P values, and Benjamini–Hochberg false-discovery rates. (C) Transcriptome-wide rank correlations and directional concordance across contrasts. (D) Representative pathway-enrichment results and cross-context comparisons, including normalized enrichment scores and leading-edge overlap. These analyses were retained as descriptive external

evidence; negative or discordant results were used to delimit the interpretation of the SERPINA1 region and were not interpreted as evidence that SERPINA1 is an upstream driver, causal regulator, or molecular mediator.

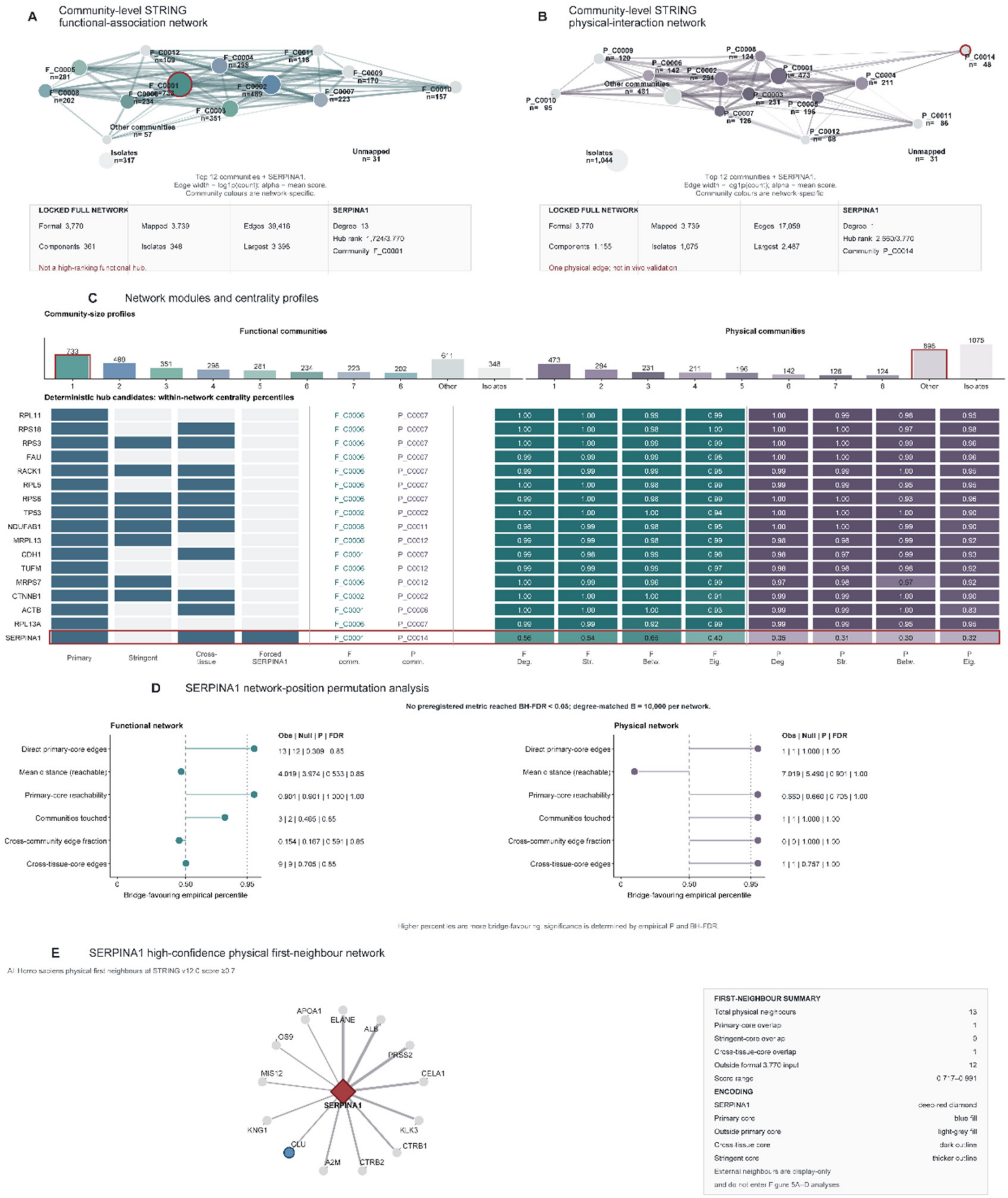

Supplementary Figure S2. STRING network position and permutation analyses of SERPINA1.

(A) Functional-association network constructed from the predefined leading-edge-derived protein set. (B) Physical-interaction network. (C) Network communities and centrality summaries. (D) Degree-matched permutation analyses evaluating the network position of SERPINA1. (E) Physical first-neighbor network of SERPINA1. Display-only external nodes were excluded from centrality, community-detection, and permutation analyses. Degree-matched permutations assessed network position conditional on degree; the STRING results were treated as contextual evidence and did not constitute experimental validation of physical interaction, causal regulation, or a SERPINA1 mechanism.
