# Supplementary material for: Genetic Associations Among Appendicular Lean Mass, Alanine Aminotransferase, and Type 2 Diabetes: Multi-Dataset Mendelian Randomization and Colocalization at the SERPINA1 Candidate Locus
Source: Genes (Basel). 2026 Sep 20;17(9):1154. doi: 10.3390/genes17091154 (PMC13606714; doi:10.3390/genes17091154)
Supplement: Supplementary file 1 [file genes-17-01154-s001.zip › Supplementary_Methods.pdf]

Supplementary Methods

Appendicular lean mass, alanine aminotransferase, type 2 diabetes, and multi-signal colocalization at the SERPINA1 candidate region: supplementary methods

S1. Data sources and analytical workflow

All analyses were conducted in R version 4.5.1. Supplementary Table S1a summarizes the GWAS identifiers, sample sizes, ancestry, source and analysis genome builds, phenotype scales, and access links.

The principal MR analyses used appendicular lean mass (ALM; ebi-a-GCST90000025; N=450,243; UK Biobank European ancestry). They also used mean alanine aminotransferase from Million Veteran Program Genomics Release 4 (MVP ALT; ALT_Mean_INT.EUR.GIA; N=406,803; European American GIA stratum). The analyses further included FinnGen R12 type 2 diabetes (T2D; 82,878 cases and 403,489 controls; total N=486,367) and body mass index (BMI; ieu-b-40; N=681,275). FinnGen R12 remained the T2D outcome throughout the pairwise MR, MVMR, and mediation analyses. ALM was interpreted as muscle mass rather than clinical sarcopenia, which also requires strength or physical-performance information.

Candidate-region phenotype and molecular-QTL colocalization used only the 2024 T2D Global Genomic Initiative European subset (T2DGGI). This subset included 242,283 cases and 1,569,734 controls (total N=1,812,017). FinnGen R12 served as a cross-cohort phenotype sensitivity dataset for phenotype colocalization. FinnGen and T2DGGI estimates were not pooled, and T2DGGI did not replace FinnGen in any MR analysis.

The MVP ALT phenotype represented participant-level mean ALT. It was adjusted for age, sex, and the first 10 principal components in a SAIGE mixed model and then inverse-normal transformed. MVP ALT effects were interpreted on the transformed GWAS beta scale rather than per unit increase in U/L. For colocalization, all datasets were standardized to GRCh37. Variants with rsIDs were mapped using the 1000 Genomes Phase 3 European reference. T2DGGI and pQTL records lacking rsIDs were matched by GRCh37 position and unordered allele set. Mapping fractions, duplicate removal, allele orientation, and LD-matrix checks were retained as explicit QC outputs.

The analysis sequence began with instrument construction and pairwise MR, followed by MR-RAPS and MR-PRESSO sensitivity analyses and one-pass RadialMR. It then included MVMR, one-pass RadialMVMR, robust and invalid-instrument MVMR sensitivity analyses, and exploratory two-step MR indirect-effect estimation. The sequence concluded with single-signal ABF and multi-signal SuSiE colocalization, transcriptome analysis, GSEA, and STRING network analysis.

Fixed random seeds were used for MR-PRESSO, SuSiE fine-mapping, the mediation bootstrap, MVMRcML data perturbations, GSEA, and network permutations. TwoSampleMR used its default 1,000 bootstrap resamples for weighted-median and weighted-mode standard errors without a fixed seed. This can cause minor run-to-run differences in secondary standard errors and P values, but not in primary IVW point estimates.

Potential sample overlap was assessed using documented cohort provenance because participant-level identifiers and exact overlap counts were unavailable. MR overlap classifications are reported in Supplementary Table S1c. A separate colocalization matrix (Supplementary Table S1d) classifies pairs involving T2DGGI as high risk when UK Biobank or MVP may occur in component cohorts. Consequently, T2DGGI results were not labeled as independent replication. FinnGen was used as a cross-cohort phenotype sensitivity source. No covariance adjustment for sample overlap was attempted.

S2. Instrument construction and harmonization for bidirectional MR

Six directions were evaluated: ALM→ALT, ALT→ALM, ALM→T2D, T2D→ALM, ALT→T2D, and T2D→ALT. The primary instrument-selection thresholds were $P<5\times10^{-9}$ for ALM and $P<5\times10^{-8}$ for ALT and T2D. BMI instruments used $P<1\times10^{-8}$ in the primary sensitivity analyses and $P<5\times10^{-8}$ in a threshold sensitivity analysis.

Candidate instruments were autosomal biallelic single-nucleotide variants with a minor-allele frequency ≥0.01, complete effect estimates, and $F=(\beta/SE)^2\geq 10$. Variants were clumped with PLINK against the 1000 Genomes European reference panel using $r^2<0.001$ within a 10,000-kb window. Variants absent from the reference panel were not used as index variants.

Exposure and outcome data were harmonized using TwoSampleMR::harmonise_data(action=2). Allele-frequency-resolvable palindromic variants were retained. Ambiguous or incompatible variants were excluded according to the mr_keep flag.

Multiplicative random-effects inverse-variance weighting (IVW) was the primary estimator. MR-Egger, the weighted median, and the weighted mode were secondary estimators. Continuous outcomes were reported on the GWAS beta scale. T2D outcomes were analyzed on the log-odds scale and reported as odds ratios with 95% confidence intervals.

S3. Pairwise-MR sensitivity analyses

Cochran's Q was calculated for the IVW and MR-Egger estimates. Directional horizontal pleiotropy was assessed using the MR-Egger intercept. Leave-one-out IVW analyses evaluated the influence of individual variants. Intercept P values were reported unadjusted and after Benjamini–Hochberg and Bonferroni correction across the six prespecified directions.

Regression dilution in MR-Egger was summarized by $I^2GX=\max[0,(QX-(K-1))/QX]$, where QX denotes inverse-variance-weighted heterogeneity in the SNP–exposure associations and K is the number of instruments.

SNP-level Steiger directionality was evaluated for all trait pairs. For comparisons involving T2D, liability-scale calculations assumed population prevalences of 5%, 10%, and 15%. The 10% scenario was used for the primary Steiger-filtered estimate.

MR-RAPS was fitted using mr.raps 0.4.3 with over.dispersion=TRUE, Huber loss, and shrinkage=FALSE. All warnings were retained. For ALM→T2D, T2D→ALM, and ALT→T2D, the overdispersion fit could not obtain a finite solution and reverted to tau2=0. These estimates were retained as sensitivity results with warnings and were not interpreted as evidence that heterogeneity or pleiotropy was absent. MR-PRESSO 1.0 used NbDistribution=20,000, SignifThreshold=0.05, and direction-specific fixed seeds.

MR-PRESSO outlier-corrected estimates and distortion tests were reported only when the global test was significant and at least one outlier was detected. P values across the six prespecified directions were adjusted using the Benjamini–Hochberg and Bonferroni procedures.

RadialMR 1.2.1 was applied to all six harmonized directions using modified second-order weights. Radial IVW outliers were identified once at a direction-specific Bonferroni threshold of 0.05/K, where K was the number of harmonized instruments. IVW estimates, Cochran's Q, and MR-Egger intercepts were recalculated after this single prespecified set was excluded. Radial MR-Egger outliers were retained as diagnostic output and were not added to the removal set. No repeated pruning was performed to force a non-significant Q statistic. Unfiltered estimates remained primary, whereas one-pass filtered estimates were treated as sensitivity analyses. Overlap between the Radial IVW and MR-PRESSO outlier sets was reported descriptively.

S4. MVMR and approximate summary-data indirect effect

The principal multivariable model included ALM and ALT as exposures and FinnGen R12 T2D as the outcome. Genome-wide significant ALM and ALT instruments were combined, jointly clumped, harmonized to a common effect-allele orientation, and restricted to complete SNP–trait associations. The original joint model contained 462 variants, whereas the prespecified one-pass RadialMVMR sensitivity set contained 450 variants.

MVMR-IVW estimation, conditional F statistics, and Q_A heterogeneity statistics used MVMR 0.4.8 throughout the analysis, supplementary tables, and reproducibility package. Multiplicative random-effects MVMR-IVW and MVMR-Egger sensitivity analyses used MendelianRandomization 0.10.0. Conditional F<10 was considered evidence of possible conditional weak-instrument bias. The joint

ALM+ALT instrument set yielded conditional F statistics above 10 for both exposures before and after one-pass RadialMVMR.

The exact covariance between SNP–ALM and SNP–ALT estimates was unavailable. Conditional strength and Q_A were therefore evaluated under the zero cross-exposure covariance assumption. They were also evaluated across the prespecified effective equicorrelation sensitivity grid ρ=−0.30, −0.10, 0, 0.10, 0.30, and 0.50. These values represent sensitivity scenarios rather than measured phenotypic or participant-level correlations.

Radial MVMR was applied once to identify exposure-specific adjusted-Q outliers after conditioning on the other exposures. The union of variants flagged at the exposure-specific Bonferroni threshold was removed once. No iterative filtering was performed to force Q_A to become non-significant. Q_A decreased by 44.2% after this step but remained strongly significant. The filtered model was therefore treated as a sensitivity analysis, and residual heterogeneity was retained as a limitation.

Robustness to invalid instruments was assessed using random-effects MVMR-IVW, MVMR-Egger, MR-Lasso, and MVMRcML-DP. MVMRcML used a fixed deterministic seed and 100 data perturbations. Convergence and the number of effective perturbations were recorded. BMI-adjusted ALM+ALT+BMI→T2D models were secondary conditional-direct-effect analyses and did not replace the two-exposure FinnGen model. Agreement in effect direction was interpreted as evidence of robustness, not as elimination of pleiotropy or heterogeneity.

For the path-specific two-step MR sensitivity[1,2] analysis, the a path (ALM→ALT) and total c path (ALM→T2D) used genome-wide significant ALM instruments. These instruments were retained in their respective pairwise harmonizations. The b path (ALT→T2D conditional on ALM) and direct c′ path used the jointly clumped ALM+ALT instrument union. All paths used MVP ALT and FinnGen R12 T2D. Repeated SNP–trait associations were aligned to the same effect allele.

The product indirect effect was defined as ab, and the decomposition contrast as c−c′. Product and difference estimators were reported separately because they need not agree in heterogeneous or pleiotropic summary-data models. No mediation proportion was calculated or exported.

Uncertainty and covariance among a, b, c, and c′ were estimated using 5,000 SNP-level parametric bootstrap replicates with shared random draws for repeated SNP–trait associations. Percentile confidence intervals and two-sided empirical P values were reported; when the empirical resolution limit was reached, values were reported as P≤0.0004 rather than as a more precise estimate. A stricter secondary diagnostic re-estimated all four paths using the same ALM-instrument subset and a delete-one-SNP jackknife. This diagnostic assessed path covariance but was not used to identify a mediation proportion.

The indirect-effect estimates were designated exploratory. The product and c−c′ estimates were not concordant: the product estimate had a small empirical P value, whereas the difference estimate was not statistically significant in either the original or post-RadialMVMR analysis. Substantial residual MVMR heterogeneity remained, and the required mediation-identification assumptions could not be verified from summary data. Accordingly, no mediation proportion was calculated, exported, or interpreted.

S4a. Candidate-region exclusion sensitivity analysis

To assess whether the MR results depended on the SERPINA1 candidate region, we performed a prespecified regional-exclusion sensitivity analysis in the GRCh37 interval chr14:94,347,262–95,347,262 (rs17580 ±500 kb). Variants within this interval were removed from the already harmonized bidirectional MR datasets and from the jointly clumped ALM+ALT multivariable MR dataset, after which the six bidirectional MR directions and the ALM+ALT→FinnGen T2D MVMR model were re-estimated. Instrument-selection thresholds, clumping criteria, allele harmonization, estimators, and the zero cross-exposure covariance assumption were unchanged. Instruments were not reselected or reclumped after regional exclusion; consequently, this analysis was treated as a sensitivity analysis and did not replace the prespecified primary models.

S5. Rationale and definition of the SERPINA1 candidate region

SERPINA1 was selected as a prior-evidence-informed candidate region and was not selected on the basis of the present colocalization results. The choice was informed by prior regional association observations, genetic evidence linking protein-altering SERPINA1 variants to liver enzymes and liver disease, and the metabolic relevance of alpha-1 antitrypsin. The study therefore evaluated a knowledge-driven candidate locus rather than conducting genome-wide locus discovery. Alternative loci were not systematically screened in the present workflow, and other shared regions cannot be excluded.

The candidate-region definition was fixed before the current colocalization calculations; "prespecified" denotes an analysis decision documented before the present colocalization run and does not imply a registered protocol.

The regional anchor was rs17580 at chromosome 14:94,847,262 (GRCh37). Analyses used a ±500-kb interval (chromosome 14:94,347,262–95,347,262, GRCh37). All ABF, SuSiE, variant-effect, and figure-source data were generated from the same canonical GRCh37 records. Effects at rs28929474 were standardized to target allele T before cross-dataset comparison.

S6. Single-signal coloc/ABF analysis

Single-signal colocalization used coloc::coloc.abf() in coloc 5.2.3. The phenotype pairs were ALM–ALT, ALM–FinnGen T2D, ALT–FinnGen T2D, ALM–T2DGGI, and ALT–T2DGGI. Molecular-QTL comparisons paired SERPINA1 cis-eQTL or cis-pQTL data with T2DGGI. ALM, MVP ALT, and molecular-QTL traits were modeled as quantitative traits. FinnGen and T2DGGI were modeled as case-control traits using their observed case fractions.

All ABF inputs used canonical GRCh37 positions and rsIDs. FinnGen was retained for the cross-cohort phenotype sensitivity analysis, whereas T2DGGI was used for the higher-powered regional analysis. The datasets were not combined. FinnGen T2D produced no eligible credible set for ALM–T2D. Therefore, this comparison was restricted to locus-level ABF evidence, and its non-estimable SuSiE result was not coded as PP.H4=0.

Variants were aligned by rsID and allele identity using direct, swapped, and strand-complement mappings. Ambiguous palindromic variants and records that failed allele, frequency, or build checks were excluded. Duplicate variants were reduced to one canonical record. ABF and SuSiE used the same standardized trait files.

For quantitative traits, sdY was estimated from minor-allele frequency, effect-size variance, and sample size when it was unavailable. For each pair, posterior probabilities were calculated for H0, H1, H2, H3, and H4. Their sum was checked numerically.

Primary priors were $p1=1\times10^{-4}$, $p2=1\times10^{-4}$, and $p12=1\times10^{-5}$. Sensitivity analyses set p12 to 1×10^{-6}, 5×10^{-6}, 1×10^{-5}, 5×10^{-5}, and 1×10^{-4}. The same grid was used for phenotype and molecular-QTL analyses.

PP.H4 was interpreted as compatibility with a shared regional association under the single-signal model. It was not interpreted as proof of a unique causal variant or causal gene. T2DGGI comparisons involving potential component-cohort overlap were labeled as overlap-sensitive supportive evidence rather than independent replication.

S7. Multi-signal SuSiE-coloc

Multi-signal analyses used coloc::runsusie(), susieR 0.14.2, and coloc::coloc.susie(). Signed LD was estimated from the 1000 Genomes Phase 3 European reference after exact SNP-order and allele-orientation matching. Each pair required matching LD row and column names, symmetry, a unit diagonal, finite entries, and at least 500 aligned variants. External LD was not treated as in-sample LD for MVP, FinnGen, T2DGGI, GTEx, or UKB-PPP.

Each trait was fine-mapped with L=10 and maxit=1,000 using pair-specific deterministic seeds. repeat_until_convergence was set to FALSE. A fit that remained non-converged after 1,000 iterations was retained as a diagnostic outcome. It was not escalated to 100,000 iterations or rescued by tuning L, priors, or coverage. For every saved fit, the analysis outputs included credible-set size, achieved and requested coverage, and minimum, mean, and median absolute within-set correlation. The

minimum absolute correlation was 0.6757–1.0000 across all formed credible sets and 0.8897–1.0000 in the key phenotype comparisons.

coloc.susie was run only when both trait fits converged and both traits produced at least one credible set. All estimable credible-set pairs were evaluated across the p12 grid in Section S6. Pairs with a fit error, non-convergence, or no credible set were classified as non-informative for signal-specific colocalization. These pairs were not assigned PP.H4=0. A limited kriging-RSS diagnostic was used to identify possible isolated allele-coding or local outlier issues[3]. It does not provide matched in-study LD or broad finite-LD mismatch correction. Within-study LD and a second European LD reference panel were not available, so external-LD signal claims remained sensitivity or overlap-sensitive evidence.

S8. SERPINA1 molecular-QTL colocalization

SERPINA1 cis-eQTLs (ENSG00000197249) were obtained from harmonized GTEx v8 resources for liver (N=208), skeletal muscle (N=702), and whole blood (N=670). Plasma AAT cis-pQTL data were obtained from the UK Biobank Pharma Proteomics Project European discovery analysis (N=33,348).

All molecular-QTL data were standardized to the rs17580±500-kb GRCh37 interval described in Section S5. eQTL records required finite beta, standard error, P value, and allele frequency. Duplicate variants were reduced to the record with the smallest P value. Molecular-QTL comparisons used T2DGGI. They were linked to the same canonical rsIDs, alleles, and signed European LD used in the phenotype analyses.

UKB-PPP pQTL records were restricted to additive, biallelic single-nucleotide variants with INFO≥0.80 and MAF≥0.01. P values were reconstructed from −log10(P), with numerical underflow bounded at the smallest positive machine value. Effects at rs28929474 were reoriented to target allele T.

All molecular-QTL–T2DGGI pairs underwent ABF analysis. Signal-specific SuSiE-coloc was reported only when both fits converged and both datasets produced credible sets. Weak or absent molecular-QTL credible sets were classified as non-informative rather than as negative evidence.

The liver and skeletal-muscle eQTL datasets did not produce eligible signal-specific comparisons. The whole-blood eQTL and plasma pQTL analyses used sample sizes of 670 and 33,348, respectively. The whole-blood eQTL and T2DGGI favored distinct signals. The plasma pQTL region contained multiple signals, and only the rs28929474 signal pair supported sharing.

ABF and SuSiE-coloc were reported separately because they assess regional and signal-specific sharing. Plasma pQTL effects may reflect abundance, secretion, protein structure, or assay binding. They were not equated with bioactive circulating AAT. UKB-PPP and T2DGGI may share UK Biobank components. Their colocalization was therefore described as supportive, overlap-sensitive evidence rather than independent replication.

S9. External transcriptome analyses

Three GEO datasets contributed five prespecified contrasts. GSE111016 skeletal-muscle RNA sequencing compared sarcopenia with control samples (20 versus 20)[4]. Genes with counts ≥10 in at least 10 samples were analyzed with the DESeq2[5] model ~age_centered+sarcopenia_status.

GSE48452 liver microarrays compared non-alcoholic steatohepatitis (n=17) and simple steatosis (n=9) separately with healthy obese controls (n=16)[6]. GSE18732 skeletal-muscle microarrays compared T2D (n=46) and impaired glucose tolerance (n=12) separately with normal glucose tolerance (n=60)[7].

Microarray analyses used GEO normalized expression matrices and log2 transformation when required. Limma models adjusted for centered age, centered BMI, sex, and group when these covariates were available in the study metadata[8].

GSE18732 was annotated with BrainArray hs133phsenst_10.0.0[9], and ENST00000355814 was the predefined SERPINA1 transcript. Each contrast reported log2 fold change, its uncertainty, nominal P value, and Benjamini–Hochberg false-discovery rate.

Effects were not pooled across tissues, platforms, or clinical contrasts. SERPINA1 expression was evaluated as external evidence and was not used to define the candidate region.

Transcriptome-wide Spearman correlations were calculated between prespecified contrasts using genes available in both comparisons. Directional concordance was summarized descriptively within and across tissues. These analyses characterized cross-context similarity. They were not interpreted as evidence of replication, causal regulation, or a shared molecular mechanism.

S10. GSEA and cross-context pathway comparison

For each contrast, all tested genes were ranked using the signed DESeq2 Wald statistic or the limma moderated t statistic. Differential-expression significance was not used to prefilter the ranked data. When several features mapped to one gene, the feature with the highest mean expression was retained.

Preranked GSEA was conducted with fgsea 1.36.2 using the fgseaMultilevel function within the established GSEA framework [10]. MSigDB 2026.1.Hs Hallmark, Reactome, and Gene Ontology Biological Process collections were tested after intersection with the ranked genes[11–13]. Gene sets containing 15–500 mapped genes were analyzed with eps=0 and scoreType="std".

Runs used deterministic seeds indexed by the prespecified contrast and library. P values were adjusted within each library and across the combined three-library result set. A combined false-discovery rate <0.05 was the primary criterion.

A pathway was considered directionally concordant when it met the primary criterion in at least two contrasts with the same normalized enrichment-score direction. Pairwise Jaccard similarity summarized leading-edge overlap. Pathway concordance and leading-edge overlap were treated as descriptive cross-context evidence. They were not used to infer that SERPINA1 was an upstream driver, mediator, or causal regulator of the enriched pathways. Supplementary Table S7 reports GSEA run summaries and all pathways meeting the prespecified global FDR threshold. It also reports cross-context pathway summaries and leading-edge overlap metrics. The complete unfiltered GSEA result matrix, including non-significant results, is provided in the reproducibility archive as all_GSEA_results.csv.gz.

S11. STRING network analysis

STRING v12.0[14] was queried for Homo sapiens (taxonomy identifier 9606) using a combined-score threshold of 0.700. The functional-association and physical-interaction networks were analyzed separately using the 3,770-protein recurrent leading-edge input set. No external interactors were added to these analyses.

A SERPINA1 physical first-neighbor network containing display-only external nodes was generated for visualization. These nodes were excluded from the centrality, community, and permutation analyses.

Degree, edge-weight strength, normalized betweenness centrality, and component-scaled eigenvector centrality were calculated for each network. Weighted shortest-path distance was defined as the reciprocal of the STRING combined score.

Leiden communities were identified separately within each connected component using resolution_parameter=1.0, n_iterations=−1, and a fixed deterministic seed. Isolated nodes remained in the analysis universe[15].

SERPINA1 network position was evaluated using 10,000 degree-matched permutations per network using a fixed deterministic seed. Six metrics were prespecified: direct edges to the primary set, mean reachable distance, primary-set reachability, communities reached, fraction of cross-community edges, and direct cross-tissue edges.

One-sided empirical P values were adjusted using the Benjamini–Hochberg procedure within each network. The permutation test evaluated network position conditional on degree. It was not interpreted as a test of biological causality.

Network-level summaries, selected hub candidates, SERPINA1-specific centrality measures, first-neighbor annotations, and degree-matched permutation results are reported in Supplementary Table S8 and Supplementary Figure S2.

S12. Computational reproducibility

Supplementary File S1 contains the analysis/ directory. The primary workflow is in analysis/primary_workflow/, and the regional-exclusion sensitivity analysis is in analysis/regional_sensitivity/run_regional_sensitivity_analysis.R. The regional outputs are provided in derived_outputs/regional_sensitivity/ and include pairwise MR, MVMR, heterogeneity, instrument-count, external-LD diagnostic, and sample-overlap classification tables; no additional figure is required. The package-level README and RUN_ORDER.md document paths, software requirements, input boundaries, and output mappings. Large or restricted source datasets and complete regional LD matrices are not redistributed.

Analysis stage	Scripts and analysis records
Input validation, canonicalization, and quality-control procedures	analysis/primary_workflow/00_preflight_analysis.R; analysis/primary_workflow/01_prepare_coloc_GRCh37.R; analysis/primary_workflow/04_build_canonical_variant_effects.R; analysis/primary_workflow/99_validate_outputs.R
Pairwise MR and sensitivity analyses	analysis/05_extract_cross_trait_and_harmonise.R; analysis/06_run_pairwise_MR.R; analysis/07_MR_RAPS_Steiger.R; analysis/08_MR_PRESSO_long_run.R; analysis/09_Steiger_filtered_MR.R; analysis/15_run_RadialMR_pairwise_sensitivity.R; analysis/12_build_MR_master_summary.R
MVMR, invalid-instrument sensitivity, and indirect effect	analysis/10_run_MVMR.R; analysis/16_run_RadialMVMR_sensitivity.R; analysis/17_post_RadialMVMR_diagnostics.R; analysis/primary_workflow/10_run_MR_mediation_same_SNP_set.R; analysis/primary_workflow/12_run_path_specific_mediation_and_MVMR_heterogeneity.R; analysis/primary_workflow/13_run_MVMR_invalid_IV_and_BMI_sensitivity.R
Phenotype ABF and SuSiE colocalization	analysis/primary_workflow/01_prepare_coloc_GRCh37.R; analysis/primary_workflow/02_run_coloc_ABF_GRCh37.R; analysis/primary_workflow/03_run_coloc_SuSiE_GRCh37.R; analysis/primary_workflow/04_build_canonical_variant_effects.R
Molecular-QTL colocalization	analysis/primary_workflow/01_prepare_coloc_GRCh37.R; analysis/primary_workflow/02_run_coloc_ABF_GRCh37.R; analysis/primary_workflow/03_run_coloc_SuSiE_GRCh37.R; analysis/primary_workflow/04_build_canonical_variant_effects.R
Transcriptome and GSEA	analysis/23_prepare_GSE111016_sarcopenia_RNAseq.R through analysis/29_integrate_SERPINA1_transcriptomic_validation.R; analysis/31_run_transcriptomic_pathway_GSEA.R; analysis/32_render_transcriptomic_pathway_figures.R

Analysis stage	Scripts and analysis records
Network analysis	analysis/34_run_STRING_PPI_analysis.R
Figure regeneration	analysis/primary_workflow/11_build_Figure2_MR_only.R; analysis/primary_workflow/15_build_styled_key_Figures3_4.R
Regional exclusion sensitivity	analysis/regional_sensitivity/run_regional_sensitivity_analysis.R; derived_outputs/regional_sensitivity/*.csv

Software versions are summarized in Supplementary Table S1b and analysis/PACKAGE_REQUIREMENTS.csv. INPUT_MANIFEST.csv records data roles and provenance. ANALYSIS_DAG.csv and OUTPUT_MAP.csv map scripts to outputs, and PACKAGE_MANIFEST_SHA256.csv records code-package checksums. The workflow writes session information, input manifests, QC tables, and machine-readable result tables for the analyses described in the manuscript.

References

1. Carter, A.R.; Sanderson, E.; Hammerton, G.; Richmond, R.C.; Davey Smith, G.; Heron, J.; Taylor, A.E.; Davies, N.M.; Howe, L.D. Mendelian Randomisation for Mediation Analysis: Current Methods and Challenges for Implementation. *Eur. J. Epidemiol.* **2021**, *36*, 465–478, doi:10.1007/s10654-021-00757-1.
2. Lin, S.-C.; Lin, S.-H.; Ge, T.; Chen, C.-Y.; Lin, Y.-F. Causal Mediation Analysis: A Summary-Data Mendelian Randomization Approach. *Stat. Med.* **2025**, *44*, e10317, doi:10.1002/sim.10317.
3. Zou, Y.; Carbonetto, P.; Wang, G.; Stephens, M. Fine-Mapping from Summary Data with the “Sum of Single Effects” Model. *PLOS Genet.* **2022**, *18*, e1010299, doi:10.1371/journal.pgen.1010299.
4. Migliavacca, E.; Tay, S.K.H.; Patel, H.P.; Sonntag, T.; Civiletto, G.; McFarlane, C.; Forrester, T.; Barton, S.J.; Leow, M.K.; Antoun, E.; et al. Mitochondrial Oxidative Capacity and NAD+ Biosynthesis Are Reduced in Human Sarcopenia across Ethnicities. *Nat. Commun.* **2019**, *10*, 5808, doi:10.1038/s41467-019-13694-1.
5. Love, M.I.; Huber, W.; Anders, S. Moderated Estimation of Fold Change and Dispersion for RNA-Seq Data with DESeq2. *Genome Biol.* **2014**, *15*, 550, doi:10.1186/s13059-014-0550-8.
6. Ahrens, M.; Ammerpohl, O.; von Schönfels, W.; Kolarova, J.; Bens, S.; Itzel, T.; Teufel, A.; Herrmann, A.; Brosch, M.; Hinrichsen, H.; et al. DNA Methylation Analysis in Nonalcoholic Fatty Liver Disease Suggests Distinct Disease-Specific and Remodeling Signatures after Bariatric Surgery. *Cell Metab.* **2013**, *18*, 296–302, doi:10.1016/j.cmet.2013.07.004.
7. Gallagher, I.J.; Scheele, C.; Keller, P.; Nielsen, A.R.; Remenyi, J.; Fischer, C.P.; Roder, K.; Babraj, J.; Wahlestedt, C.; Hutvagner, G.; et al. Integration of microRNA Changes in Vivo Identifies Novel Molecular Features of Muscle Insulin Resistance in Type 2 Diabetes. *Genome Med.* **2010**, *2*, 9–26, doi:10.1186/gm130.
8. Ritchie, M.E.; Phipson, B.; Wu, D.; Hu, Y.; Law, C.W.; Shi, W.; Smyth, G.K. Limma Powers Differential Expression Analyses for RNA-Sequencing and Microarray Studies. *Nucleic Acids Res.* **2015**, *43*, e47–e47, doi:10.1093/nar/gkv007.
9. Dai, M. Evolving Gene/Transcript Definitions Significantly Alter the Interpretation of GeneChip Data. *Nucleic Acids Res.* **2005**, *33*, e175–e175, doi:10.1093/nar/gni179.
10. Subramanian, A.; Tamayo, P.; Mootha, V.K.; Mukherjee, S.; Ebert, B.L.; Gillette, M.A.; Paulovich, A.; Pomeroy, S.L.; Golub, T.R.; Lander, E.S.; et al. Gene Set Enrichment Analysis: A Knowledge-Based Approach for Interpreting Genome-Wide Expression Profiles. *Proc. Natl. Acad. Sci.* **2005**, *102*, 15545–15550, doi:10.1073/pnas.0506580102.
11. Liberzon, A.; Birger, C.; Thorvaldsdóttir, H.; Ghandi, M.; Mesirov, J.P.; Tamayo, P. The Molecular Signatures Database Hallmark Gene Set Collection. *Cell Syst.* **2015**, *1*, 417–425, doi:10.1016/j.cels.2015.12.004.
12. Milacic, M.; Beavers, D.; Conley, P.; Gong, C.; Gillespie, M.; Griss, J.; Haw, R.; Jassal, B.; Matthews, L.; May, B.; et al. The Reactome Pathway Knowledgebase 2024. *Nucleic Acids Res.* **2024**, *52*, D672–D678, doi:10.1093/nar/gkad1025.

13. The Gene Ontology Consortium; Carbon, S.; Douglass, E.; Good, B.M.; Unni, D.R.; Harris, N.L.; Mungall, C.J.; Basu, S.; Chisholm, R.L.; Dodson, R.J.; et al. The Gene Ontology Resource: Enriching a GOld Mine. *Nucleic Acids Res.* **2021**, *49*, D325–D334, doi:10.1093/nar/gkaa1113.
14. Szklarczyk, D.; Nastou, K.; Koutrouli, M.; Kirsch, R.; Mehryary, F.; Hachilif, R.; Hu, D.; Peluso, M.E.; Huang, Q.; Fang, T.; et al. The STRING Database in 2025: Protein Networks with Directionality of Regulation. *Nucleic Acids Res.* **2025**, *53*, D731–D737, doi:10.1093/nar/gkae1113.
15. Traag, V.A.; Waltman, L.; Van Eck, N.J. From Louvain to Leiden: Guaranteeing Well-Connected Communities. *Sci. Rep.* **2019**, *9*, 5233, doi:10.1038/s41598-019-41695-z.
